# CircZBTB46, a promising therapeutic target in crizotinib resistant ALK-positive T lymphomas

**DOI:** 10.1038/s41375-026-03018-w

**Published:** 2026-06-26

**Authors:** Loélia Babin, Elissa Andraos, Steffen Fuchs, Chloe Bessière, Lola Colras, Sandra Dailhau, Cathy Quelen, Cindy Pinto, Iyad Daoudi, Romain Pfeifer, Ahmed Zamani, Marina Bousquet, Stéphane Pyronnet, Christine Gaspin, Laurence Lamant, Fabienne Meggetto

**Affiliations:** 1 Univ Toulouse, CNRS, INSERM, CRCT (Cancer Research Center of Toulouse), UMR-1037, UMR-5071, Institut Universitaire du Cancer, Toulouse, France; 2 Laboratoire d’Excellence Toulouse Cancer-TOUCAN, Toulouse, France; 3 Department of Pediatric Oncology and Hematology, Charité - Universitätsmedizin, Corporate Member of Freie Universität Berlin and Humboldt-Universität, Berlin, Germany; 4 German Cancer Consortium (DKTK), Partner Site Berlin, a Partnership Between DKFZ and Charité-Universitätsmedizin, Berlin Institute of Health at Charité – Universitätsmedizin Berlin, Berlin, Germany; 5 INRAE, BioinfOmics, GenoToul Bioinformatics Facility, Université de Toulouse, Castanet-Tolosan, Toulouse, France; 6 INRAE, MIAT, Université de Toulouse, Castanet-Tolosan, Toulouse, France

**Keywords:** Haematological cancer, T-cell lymphoma

## Abstract

Circular RNAs (circRNAs) are increasingly recognized as functional non-coding transcripts with oncogenic potential. Here, a comprehensive analysis of circRNA expression in primary ALK(+) anaplastic large-cell lymphoma (ALK(** + **) ALCL) is presented. Integrated transcriptomic profiling revealed that aberrant expression of circZBTB46 and of its linear host transcript, normally restricted to dendritic cells, is exclusive to ALK(+) lymphoma cells and driven by the oncogenic NPM1::ALK/STAT3 axis. Functional studies showed that circZBTB46, unlike its protein-coding counterpart, promotes resistance to the ALK inhibitor crizotinib. Silencing circZBTB46 restored crizotinib sensitivity in resistant ALCL cells both in vitro and in vivo. Transcriptomic analyses identified PIP5K1C as a downstream effector regulated through a competitive endogenous RNA mechanism in which circZBTB46 acts as a sponge to miR-25-3p, alleviating its repression of PIP5K1C. These findings uncover a previously unrecognized mechanism of drug resistance in ALK(** + **) ALCL and establish circZBTB46 as a promising therapeutic target.

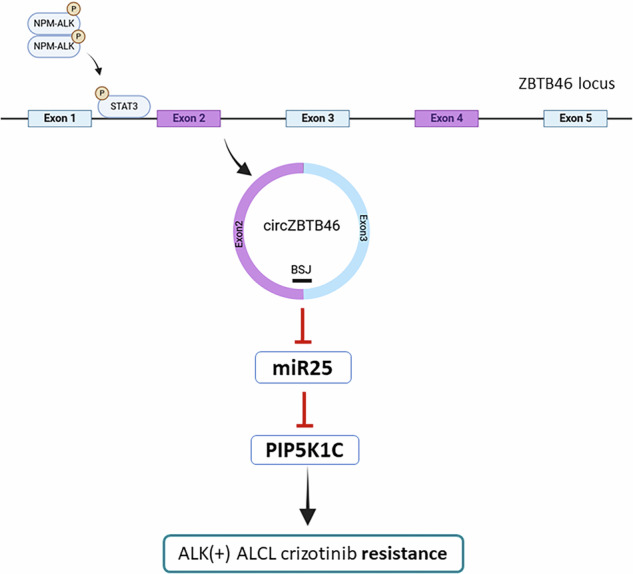

## Introduction

The purpose of this study was to investigate circRNAs in the specific context of ALK( + ) ALCL. Anaplastic large-cell lymphoma (ALCL) is a rare and aggressive peripheral T-cell non-Hodgkin lymphoma (NHL) belonging to CD30^+^ lymphoproliferative disorders [1]. ALCL is divided into two subtypes based on the expression of the anaplastic lymphoma kinase (ALK) [2]. The ALK(+) subtype primarily affects children and young adults. In approximately 80–85% of ALK(+) cases, the t(2;5)(p23;q35) chromosomal translocation is present, driving the fusion of the intracytoplasmic domain of ALK, on chromosome 2p23, with the N-terminal portion of nucleophosmin (NPM1), on chromosome 5q35 [3]. This NPM1::ALK fusion protein constitutively activates key oncogenic signaling pathways, including RAS-ERK, JAK3-STAT3 and PI3K-Akt, thereby driving cancer cell proliferation, differentiation and survival [4].

In ALK( + ) ALCL, current first-line multidrug chemotherapy (CT) regimens achieve a progression-free survival of approximately 70% at ten years after diagnosis. Crizotinib, the first ALK-targeted tyrosine kinase inhibitor (TKI), was introduced for the treatment of ALK( + ) ALCL refractory or relapsing (R/R) after CT. However, the clinical efficacy of crizotinib has been hampered by the emergence of resistance. The prognosis for R/R patients remains poor, highlighting the need for a better understanding of the aggressive behavior of ALCL and the development of novel prognostic markers to identify high-risk patients at an early stage [5, 6]. Previous studies have disclosed the important role of small noncoding RNAs, particularly microRNAs (miRNAs), in ALCL [7]. We and others have shown that specific miRNAs are associated with an increased risk of relapse in ALK( + ) ALCL, after CT or crizotinib treatment [8–13]. Circular RNAs (circRNAs) are a recently recognized class of stable and conserved noncoding RNAs characterized by a covalently closed loop structure lacking 5′ and 3′ ends. Predominantly localized in the cytoplasm, circRNAs act as molecular sponges for microRNAs and RNA-binding proteins, thereby modulating post-transcriptional gene regulation. They are highly stable and often exhibit tissue- or stage-specific expression patterns. Dysregulation of circRNAs has been shown to contribute to the pathophysiology of several solid tumors and a range of hematological malignancies [14–16]. A few reports have addressed the implication of circRNAs in peripheral T-cell NHL such as T-cell lymphoblastic lymphoma [15, 17], but to date, no studies appear to have examined their role in ALK( + ) ALCL. Deciphering the functions and mechanisms of circRNAs in ALK( + ) ALCL could lead to the identification of innovative biomarkers development of therapeutic

## Results

### Aberrant expression of circRNA and mRNA *ZBTB46* in NPM1::ALK(+) lymphoma cells

This work was initiated by investigating genome-wide circRNA expression profiles in ALK( + ) ALCL via various approaches.

**Fig. 1:**
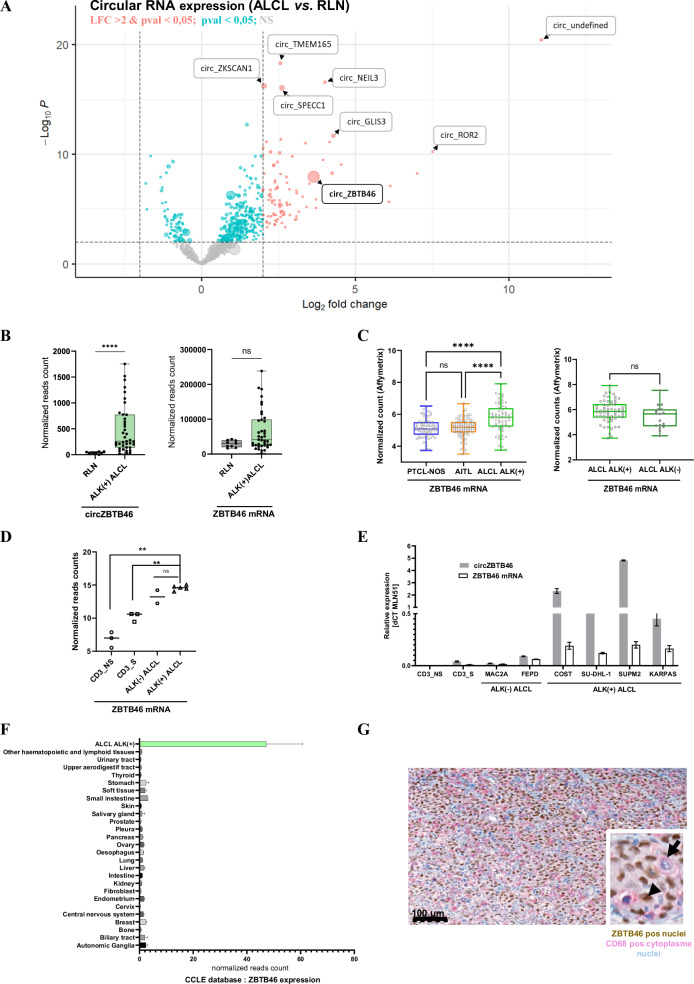
Expression of circZBTB46 and ZBTB46 mRNAs in ALK( + ) ALCL primary tumors and normal tissues. **A** Volcano plot of circular RNA expression comparing ALK( + ) ALCL primary biopsies (*n* = 39) and healthy tissues (reactive lymph nodes, RLN, *n* = 9). Size of each point is proportional to the mean expression of the corresponding circular RNA (**B**) expression of circZBTB46 (left) and ZBTB46 mRNA (right) assessed by RNA-Seq in ALK( + ) ALCL primary samples versus RLN. **C** ZBTB46 mRNA expression (RMA) from microarray datasets in ALK( + ) ALCL (*n* = 61), angioimmunoblastic T-cell lymphoma (AITL, *n* = 83), peripheral T-cell lymphoma not otherwise specified (PTCL-NOS, *n *= 71) and ALK(-) ALCL (*n* = 17) primary biopsies. **D** ZBTB46 mRNA expression (log2 of the transcript count per million (lenghScaled TPM) from RNA-Seq data in five ALK( + ) ALCL cell lines (KARPAS-299, SU-DHL-1, SUP-M2, PIO, COST), two ALK(-) ALCL cell lines (FEPD, MAC-2A) and CD3(+) lymphocytes stimulated (S, *n* = 3) or not (NS, *n* = 3). **E** Quantitative real-time PCR (RT‒qPCR) analysis of circZBTB46 and ZBTB46 mRNAs in the same cell types. MLN51 was used as an internal control. Values are expressed as 2^(–Δ)Ct relative ratios. Experiments were performed at least in triplicate. Statistical significance was assessed via an unpaired two-tailed Student’s t test with Welch’s correction: *P* < 0.01 (**), *P* < 0.001 (***), *P* < 0.0001 (****), ns = not significant. Data are expressed as means ± SD. **F** ZBTB46 mRNA expression across malignancies in the Cancer Cell Line Encyclopedia (CCLE) from RNAseq dataset. **G** Representative immunohistochemical image of an ALK( + ) ALCL primary tumor showing ZBTB46 (brown, arrowhead) and CD68 (red, arrow) expression. Cell nuclei were counterstained with hematoxylin (blue). Original magnification, ×24.6.

First, a ribo-minus RNA sequencing (RNAseq) dataset, previously generated from a cohort of 39 ALK( + ) ALCL biopsies, was analyzed, with 9 reactive lymph nodes (RLNs) as controls [18] (Supplementary Table S1). A circRNA expression landscape was established in ALK( + ) ALCL (log2Fold Change ≥2 and a *P* value < 0.05, Supplementary Table S2 and Fig. 1A). Among upregulated circRNAs, circZBTB46 displayed the highest expression (L2FC: 3.6, base mean expression 498.3; Fig. 1A). This circRNA stood out for several reasons. First, using long-read Oxford Nanopore RNA-seq, our group has already identified circZBTB46 as one of the most abundant circRNAs expressed in four human ALK( + ) ALCL cell lines [19]. Second, the expression of its host gene, *ZBTB46* (also known as BTBD4, zDC, BZEL, RINZF, and ZNF340) is a transcription factor belonging to the BTB-ZF (broad complex, tramtrack, bric-à-brac, and zinc finger) family of transcription repressors and considered to be restricted to human progenitor and conventional dendritic cells but has not been reported in T-lymphocytes [20–22]. Finally, using the RNAseq dataset, it was found that both *ZBTB46* circRNA is undetectable in RLN (Fig. 1B). In a second step, published microarray data from peripheral T-cell lymphomas (PTCL) [18] were analyzed. *ZBTB46* mRNA levels were compared in samples from ALCL (*n *= 61), PTCL-not-otherwise specified (PTCL-NOS, *n* = 71), angioimmunoblastic T-cell lymphoma (AITL, *n* = 83) and ALK negative anaplastic large cell lymphoma (ALK(-); *n* = 17) [23, 24]. The linear *ZBTB46* transcript was found to be significantly upregulated in ALK( + ) ALCL compared with other PTCL subtypes (Fig. 1C). Notably, no significant difference in ZBTB46 mRNA expression was observed between ALK-positive (ALK + ) and ALK-negative (ALK-) samples within ALCL cases (Fig. 1C).

Next, expression levels of *ZBTB46* RNA were analyzed in four ALK( + ) ALCL cell lines (COST, SU-DHL-1, SUPM2 and KARPAS-299), two ALK(-) cell lines (FEPD and Mac2a) as well as in stimulated (S) and non-stimulated (NS) normal CD3^+^ lymphocytes (*n* = 3 donors). Both RNAseq (Fig. 1D) and RT‒qPCR (Fig. 1E) analyses revealed no significant difference in linear ZBTB46 mRNA expression between ALK(+) and ALK( − ) ALCL cell lines, whereas both ALK(+) and ALK(−) cell lines showed a strong upregulation of *ZBTB46* circRNA compared with ALK(-) cell lines and normal CD3+ lymphocytes, independently of stimulation.

In addition, RT-qPCR analysis showed a marked overexpression of circZBTB46 in ALK( + ) ALCL cell lines compared with controls (Fig. 1E). These findings were confirmed at the protein level via Western blotting (Supplementary Fig. 1A).

Using the Cancer Cell Line Encyclopedia (CCLE) database portal [25] we found again that expression of the linear *ZBTB46* transcript was far the highest in ALK( + ) ALCL cell lines among cancer cell lines (Fig. 1F).

Finally, immunohistochemistry on primary biopsies from 19 ALK(+) and 8 ALK( − ) ALCL cases revealed that ZBTB46 protein was strongly and uniformly expressed in 100% of tumor cells in all ALK(+) cases (Fig. 1G), whereas ALK(−) tumor cells displayed more heterogeneous and generally moderate staining (Supplementary Fig. 1B), consistent with our qRT-PCR results (Fig. 1C–E). No ZBTB46 protein was detected in CD68+ cells (macrophages and dendritic cells) within the tumor biopsies (Fig. 1G). In contrast, reactive lymph nodes; i.e non-tumoral tissue, displayed moderate ZBTB46 staining in sinusoidal macrophage-like cells and in endothelial cells, consistent with previous reports on normal human lymphoid tissue [26, 27].

Collectively, these findings show that both circZBTB46 and its linear counterpart are strongly expressed in all tumor cells of ALK( + ) ALCL, highlighting their predominant association with this lymphoma subtype.

### Basic information and characteristics of circZBTB46 in ALK(+) lymphoma cells

**Fig. 2:**
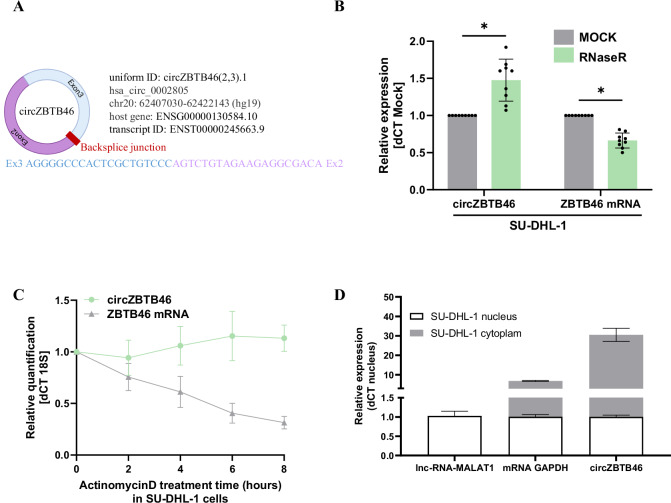
Characterization of circZBTB46 in the SU-DHL-1 ALK( + ) ALCL cell line. **A** Schematic representation of the circZBTB46 structure with Sanger sequencing of the back-splice junction. **B** Quantitative real-time PCR (RT‒qPCR) analysis of circZBTB46 and ZBTB46 mRNA expression in cells treated or not with RNase R, a 3′-5′ exonuclease that degrades linear RNAs but spares circular RNAs. **C** RT‒qPCR analysis after treatment with actinomycin D, an inhibitor of RNA synthesis, to assess transcript stability. 18S rRNA was used as an internal control. Expression values are displayed as 2-∆∆Ct relative ratios. **D** Subcellular localization of circZBTB46 and ZBTB46 mRNAs determined by cellular fractionation. MALAT1 and GAPDH mRNAs were used as nuclear and cytoplasmic controls, respectively. All experiments were performed in triplicate. Statistical significance was assessed via an unpaired two-tailed Student t test with Welch correction: *P* < 0.05 (*). Data are expressed as means ± SEM.

As previously reported [19], circZBTB46 is a 1255-nucleotide-long circRNA produced by backsplicing exons 2 and 3 of the linear *ZBTB46* transcript (ENST00000245663.9, Fig. 2A and Supplementary Table S3). CircZBTB46 is listed in circBase and circBank as hsa_circ_0002805, and mapped to the genomic coordinates chr20:62407030-62422143 (hg19). Moreover, it is annotated as circZBTB46(2,3).1 according to the standardized nomenclature for eukaryotic circular RNAs [28]. The circular nature of circZBTB46 in the ALK( + ) ALCL cell line SU-DHL-1, was confirmed by resistance to exonuclease digestion (RNase R), unlike linear *ZBTB46* mRNA (Fig. 2B). Furthermore, upon transcription arrest by actinomycin D, circZBTB46 remained stable for 8 h while its linear counterpart declined rapidly (Fig. 2C). Finally, subcellular fractionation revealed that circZBTB46 was predominantly localized in the cytoplasm (Fig. 2D).

Collectively, these results support that circZBTB46 is a bona fide circular RNA transcript with a cytoplasmic localization.

### The NPM1::ALK/STAT3 axis is responsible for the aberrant accumulation of circRNA and linear *ZBTB46* transcripts in ALK(+) lymphoma cells

**Fig. 3:**
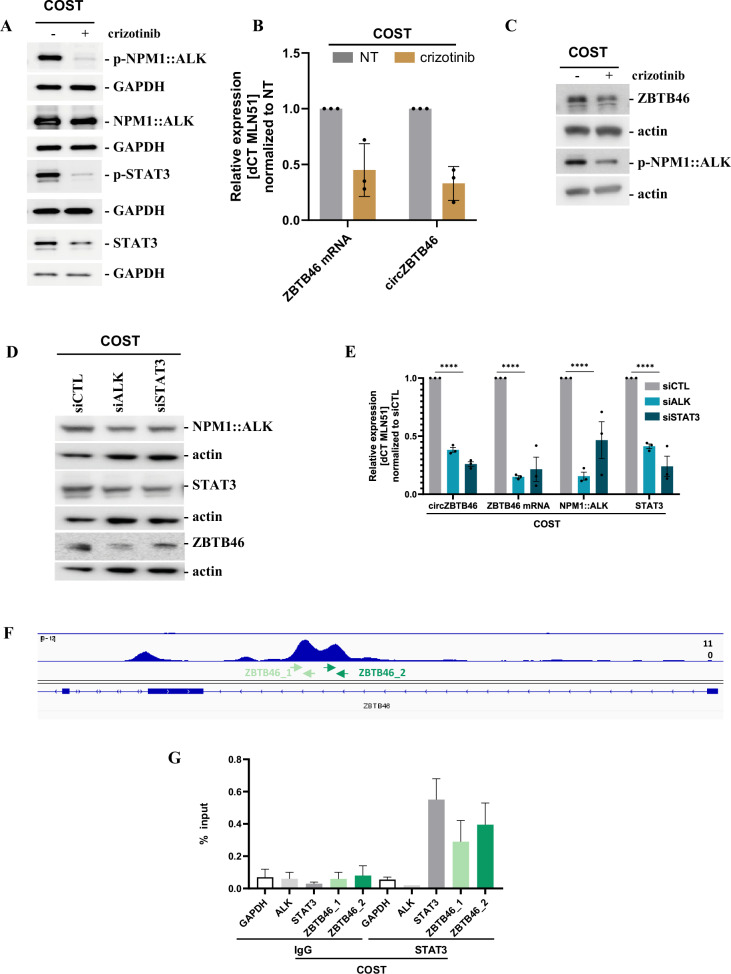
Regulation of *circZBTB46* and *ZBTB46* expression by ALK and STAT3 signaling in ALK( + ) ALCL. **A** Western blot analysis of total and phosphorylated ALK (ALK, p-ALK) and STAT3 (STAT3, p-STAT3) protein levels in COST cells treated for 48 h with crizotinib (400 μM). **B** RT‒qPCR analysis of *circZBTB46* and *ZBTB46* mRNA levels following crizotinib treatment (Crizo). **C** Western blot analysis of ZBTB46 and p-ALK protein expression under the same conditions. **D** Western blot and (**E**) RT‒qPCR analysis of ZBTB46, ALK, and STAT3 expression in COST cells transfected for 48 h with control siRNA (siCTL), ALK-targeting siRNA (siALK), or STAT3-targeting siRNA (siSTAT3). GAPDH or actin were used as a loading controls. MLN51 served as an internal control for RT‒qPCR. mRNA expression values are shown as 2-∆∆Ct relative ratios. Experiments were performed at least in triplicate. **F** STAT3 ChIP-seq data from the JB6 cell line showing enrichment of STAT3 at the *ZBTB46* locus (data from GSE117164; [30]). **G** ChIP‒qPCR analysis using two primer pairs (ZBTB46_1 and ZBTB46_2) showing STAT3 enrichment at the *ZBTB46* promoter in COST cells, represented as % input. STAT3 was a positive control and ALK and GAPDH were used as negative controls. IgG was used as a nonspecific binding control. Experiments were performed at least in triplicate. Representative Western blots from three independent experiments are shown. Statistical significance was assessed via an unpaired two-tailed Student t test with Welch correction: *P* < 0.05 (*), *P* < 0.0001 (****). Data are expressed as means ± SEM.

The consistent upregulation of both circRNA and linear *ZBTB46* transcripts in NPM1::ALK(+) cell lines and patient primary biopsies suggests that the NPM1::ALK fusion protein plays a central role in driving this overexpression. This was investigated by analyzing *ZBTB46* linear mRNA levels in T-cells ectopically expressing NPM1::ALK, using two complementary models, i) primary human CD4^+^ T-lymphocytes transduced with a lentiviral vector encoding NPM1::ALK (M1 model) [29] and ii) T-cells engineered to carry the canonical t(2;5)(p23;q35) translocation via CRISPR-Cas9 genome editing (ALKIma1 model) [19]. In both models, previously generated RNAseq datasets were used to demonstrate robust induction of *ZBTB46* mRNA following immortalization and transformation of CD4^+^ T-cells by the *NPM1::ALK* oncogene (Supplementary Fig. 2A and 2B). To further determine whether the tyrosine kinase activity of NPM1::ALK is required for this regulation, the ALK( + ) ALCL cell line COST was treated with crizotinib. TKI treatment indeed efficiently inhibited ALK kinase activity, as indicated by the loss of NPM1::ALK autophosphorylation (p-ALK). A concomitant reduction in STAT3 activation was evidenced by decreased p-STAT3 levels in Western blot (Fig. 3A). RT‒qPCR and Western blot further demonstrated a significant downregulation of both circZBTB46 and linear *ZBTB46* transcripts (Fig. 3B), as well as reduced ZBTB46 protein levels. (Fig. 3C). siRNAs against *NPM1::ALK* (siALK) or *STAT3* (siSTAT3) were then used in two ALK( + ) ALCL cell lines, COST and SUP-M2. Efficient knockdown of NPM1::ALK and STAT3 was confirmed at the protein level (Fig. 3D and Supplementary Fig. 2C). RT‒qPCR analysis demonstrated that knockdown of either *NPM1::ALK* or *STAT3* strongly decreased circZBTB46 and linear *ZBTB46* expression in both cell lines (Fig. 3E and Supplementary Fig. 2D), indicating that NPM1::ALK-dependent STAT3 signaling is critical for driving *ZBTB46* accumulation and circZBTB46 production. Whether STAT3 binds directly to the *ZBTB46* locus was investigated by analyzing publicly available STAT3 ChIP-seq datasets (GSE117164) generated from two ALK( + ) ALCL cell lines (JB6 and SU-DHL-1) exposed or not to crizotinib [30]. As a positive control, strong STAT3 binding peaks were observed at the regulatory regions of *IRF4*, a known direct STAT3 target in ALK( + ) ALCL cells [31]. Two main STAT3 binding peaks were detected in the *ZBTB46* locus in untreated cells, whereas TKI treatment significantly reduced STAT3 occupancy at these sites, consistent with the loss of STAT3 activation (Supplementary Fig. 3A). ChIP‒qPCR, using a STAT3-specific antibody in the ALCL cell lines SUP-M2 and COST, and two independent primer pairs within the *ZBTB46* locus, allowed for precise quantifications of STAT3 binding (ZBTB46_1 and ZBTB46_2, Fig. 3F). Enrichment of STAT3 binding at its two binding sites was confirmed in the *ZBTB46* locus (Fig. 3G and Supplementary Fig. 3B).

Together, these data support a direct regulatory role of the NPM1::ALK/STAT3 axis in the aberrant accumulation of linear and circRNA *ZBTB46* transcripts in ALK ( + ) ALCL cells.

### CircZBTB46 promotes crizotinib resistance in ALK(+) lymphoma cells

**Fig. 4:**
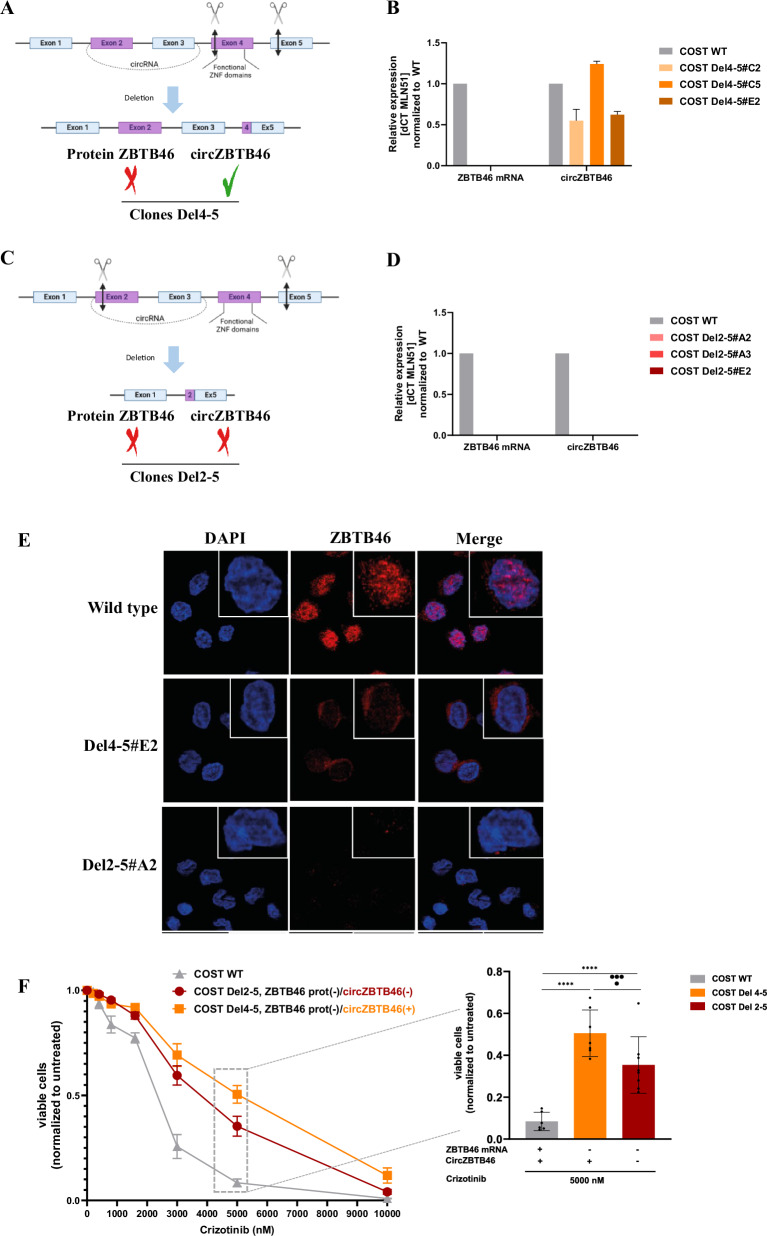
Generation and characterization of ZBTB46 CRISPR/Cas9 knockout models. **A** Schematic representation of the genomic deletion between exons 4 and 5 (Del4–5). **B** Relative expression levels of *circZBTB46* and *ZBTB46* mRNAs determined by RT‒qPCR in COST Del4–5 clones. **C** Schematic representation of the genomic deletion between exons 2 and 5 (Del2–5). **D** Relative expression levels of *circZBTB46* and *ZBTB46* mRNAs determined by RT‒qPCR in COST Del2–5 clones. *MLN51* was used as an internal control for RT‒qPCR. Data are expressed as 2-∆∆Ct relative values. All experiments were performed with at least three biological replicates. **E** Immunofluorescence analysis of ZBTB46 protein expression in parental (wild-type) COST cells and Del4–5#A2 and Del2–5#E2 clones. Nuclei were counterstained with DAPI (blue). Original magnification, 63×. **F** Cell viability was assessed by Annexin V-Pacific blue/PI staining (flow cytometry) in parental COST cells and Del4-5 and Del2-5 clones treated with increasing concentrations of crizotinib (1, 2, 3, 5 and 10 µM) for 48 h.

**Fig. 5:**
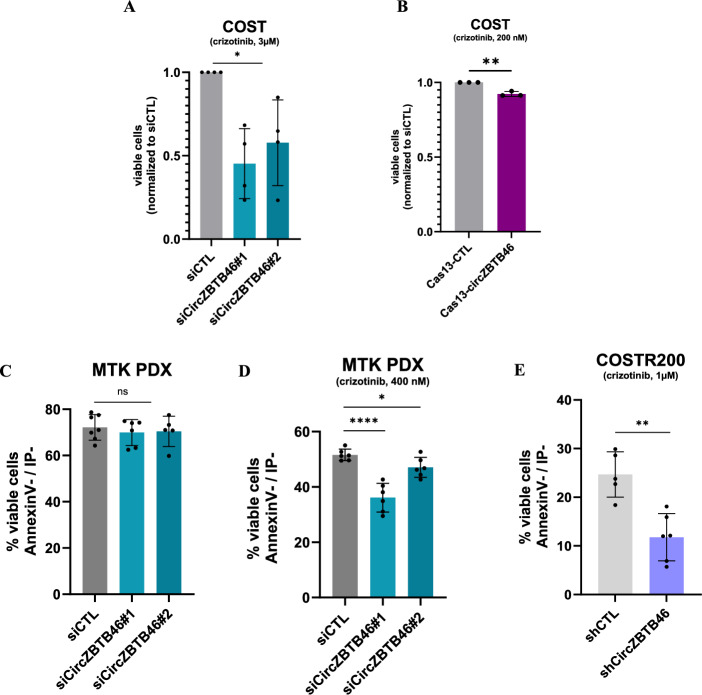
Functional effects of circZBTB46 depletion and sensitivity to crizotinib. **A** Viability (Annexin V-Pacific Blue/PI flow cytometry) of COST cells transfected with siCircZBTB46#1 or #2 and subsequently treated with crizotinib (3 µM, 48 h). **B** Viability (Annexin V-Pacific Blue/PI flow cytometry) of COST cells transduced as described in (Supplementary Fig. 5A) and treated with crizotinib (200 nM) for 7 days. Cell viability assessed by Annexin V-Pacific Blue/PI staining (flow cytometry) in crizotinib-resistant cells established from MTK PDX-derived cells transfected with siCircZBTB46#1 or #2, without treatment (**C**) or after crizotinib exposure (**D**) (400 nM, 48 h). **E** Viability of COSTR200 cells determined by Annexin V/PI staining (flow cytometry) after transduction with circZBTB46 shRNA and subsequent treatment with crizotinib (1000 nM, 7 days). MLN51 was used as an internal control for RT‒qPCR. Expression data are displayed as 2-∆∆Ct relative values. Statistical significance was assessed via an unpaired two-tailed Student t test with Welch correction: *P* < 0.05 (*), *P* < 0.01 (**), *P* < 0.0001 (****), ns = not significant. Data are expressed as means ± SEM.

To dissect the respective roles of circZBTB46 and its linear mRNA counterpart, CRISPR-Cas9 knockout models were generated in the COST cell line. Since both the zinc finger domain (ZNF) and nuclear localization signal (NLS) of ZBTB46 are encoded by exon 4, two guide RNAs targeting exons 4 and 5 were used, to induce loss of function of the ZBTB46 protein (Fig. 4A). Deletion of exon 4 in three independent clones, Del4-5#C2, #C5 and #E2, was confirmed via genomic PCR (Supplementary Fig. 4A) and RT‒qPCR (Fig. 4B). This strategy resulted truncated ZBTB46 protein expression, as confirmed in Western blot, using an antibody directed against an epitope encoded by exon 2 (Supplementary Fig. 4B). Additional clones lacking both the functional protein (i.e. mRNA) and circZBTB46 were generated using guide RNAs targeting exons 2 and 5 (Fig. 4C), resulting in the deletion of exons 2 to 4. DNA PCR and RT‒qPCR confirmed successful deletion in three clones, Del2-5#A2, #A3, and #E2 (Fig. 4D and Supplementary Fig. 4A). As expected, these clones exhibited loss of both ZBTB46 protein (Supplementary Fig. 4B) and circRNA (Fig. 4D). In immunofluorescence, the full-length ZBTB46 transcription factor was localized in the nucleus of parental wild-type COST cells (Fig. 4E), while it was in the cytoplasm of Del4-5 clones (Supplementary Fig. 4C) and absent in Del2-5 clones (Fig. 4E and Supplementary Fig. 4B, C). The proliferation and survival rates of parental Del4-5 and Del2-5 clones were comparable (Supplementary Fig. 4D, E). However, after 48 h of treatment with crizotinib, both Del4-5 and Del2-5 clones exhibited increased survival compared with parental wild-type cells (Fig. 4F). Importantly, Del2-5 clones, lacking circZBTB46 expression, presented significantly less TKI resistance than clones retaining this circRNA (Del4-5). To gain deeper insight into this observation, selective siRNA downregulation of circZBTB46 in the COST and KARPAS-299 cell-lines, was designed to target the back-splice junction and specifically deplete circZBTB46 without affecting the linear *ZBTB46* transcript (siCircZBTB46#1 and #2; Supplementary Fig. 5A). After 48 h of crizotinib treatment, both cell lines transfected with circZBTB46-targeting siRNAs exhibited reduced survival compared with control siRNA-treated cells (Fig. 5A and Supplementary Fig. 5B), supporting the involvement of circZBTB46 in crizotinib resistance in ALK( + ) ALCL.

Based on these results, a CRISPR/Cas13 lentiviral system was used to stably suppress the expression of circZBTB46 (Cas13_circZBTB46) in the COST, SUPM2 and PIO cell lines. This approach resulted in an approximately 50% reduction in circZBTB46 levels without affecting *ZBTB46* mRNA or protein expression (Supplementary Fig. 5C, D). Stable circZBTB46 knockdowns were used to assess the effect of prolonged crizotinib exposure (7 days, 100 or 200 nM). Compared with controls (Cas13_CTL), the three transduced cell lines (Cas13_circZBTB46) exhibited significantly reduced viability upon treatment (Fig. 5B and Supplementary Fig. 5E), indicating increased sensitivity to TKI.

### Loss of circZBTB46 restores the sensitivity of resistant ALK( + ) ALCL cells to crizotinib

To explore the therapeutic potential of targeting circZBTB46 in crizotinib-resistant ALK( + ) ALCL, siRNA-mediated inactivation of circZBTB46 was performed in MTK PDX-derived cells, obtained from a patient with crizotinib-refractory disease [32]. Two independent siRNAs targeting circZBTB46 were used to ensure specificity of the observed effects (Supplementary Fig. 5F). Silencing circZBTB46 did not affect cell viability in the absence of treatment (Fig. 5C). However, after 48 h of crizotinib exposure, transfected cells showed increased sensitivity to crizotinib compared with those treated with control siRNAs (Fig. 5D). We next generate stable circZBTB46-knockdown cell lines via shRNA lentiviral transduction (shCircZBTB46). Owing to the low transduction efficiency of MTK PDX-derived cells, a previously established crizotinib-resistant derivative of the COST cell line (COSTR200), was used [33]. The effective and specific downregulation of circZBTB46 was confirmed (Supplementary Fig. 5G). As expected, circZBTB46 knockdown sensitized COSTR200 cells to crizotinib in vitro (Fig. 5E).

### CircZBTB46 induces crizotinib resistance by modulating PIP5K1C gene expression in ALK( + ) ALCL

**Fig. 6:**
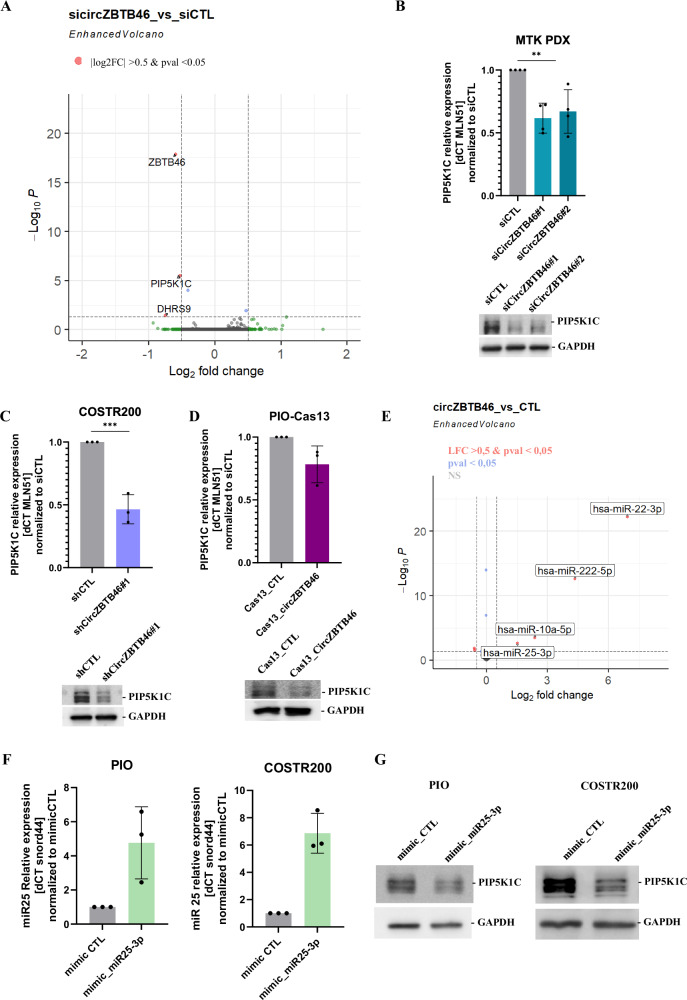
circZBTB46 modulates PIP5K1C by sponging miR-25. **A** Volcano plot showing differentially expressed genes in crizotinib-resistant cells established from MTK PDX-derived cells transfected with control siRNA (siCTL) or siRNAs targeting circZBTB46 (siCircZBTB46#1 and #2). **B** Expression of PIP5K1C at the mRNA (top panel, RT‒qPCR) and protein (bottom panel, Western blot) levels in MTK-PDX ALK( + ) ALCL cells after circZBTB46 siRNA transfection (48 h). **C** Expression of PIP5K1C at the mRNA (top panel) and protein (bottom panel) levels in stable COSTR200 cells expressing either control shRNA (CTL) or shRNA targeting circZBTB46, as assessed by RT‒qPCR and Western blot, respectively. **D** Expression of PIP5K1C in PIO ALK( + ) ALCL cells stably expressing Cas13 with control gRNA (CTL) or gRNA targeting circZBTB46, as determined by RT‒qPCR (top panel) and immunoblotting (bottom panel), respectively. **E** Volcano plot showing in the COST cell line differentially enriched RNAs in circZBTB46 pull-down compared to control, highlighting specific microRNAs enriched in the circZBTB46 fraction. **F** Relative expression of miR25-3p in crizotinib-sensitive (PIO) and resistant COSTR200 cells transfected with a negative control mimic (mimicCTL) or mimic_25-3p measured by RT-qPCR. Snord44 was used as an internal control. Data are shown as 2-∆∆Ct relative values. **G** Western blot analysis of PIP5K1C protein levels in PIO and COSTR200 cells transfected with a miR_25-3p mimic or control mimic. GAPDH served as a loading control. Experiments were performed in triplicate. Data are presented as mean ± SEM. Statistical significance was determined using an unpaired two-tailed Student *t* test with Welch correction: *P* < 0.05 (*), *P* < 0.001 (**), ns = not significant.

To identify molecular pathways regulated by circZBTB46, RNA-Seq was performed after the transfection of two independent siRNAs targeting circZBTB46 in crizotinib-resistant MTK PDX-derived cells. Both siRNAs induced comparable transcriptional changes, particularly downregulation of PIP5K1C and DHRS9 ( | log₂Fold Change | ≥ 0.5, *P* < 0.05) (Fig. 6A and Supplementary Table S4). The downregulation of PIP5K1C was validated by RT‒qPCR and Western blotting, confirming reduced expression at both mRNA and protein levels in all cellular models previously described (Fig. 6B–D and Supplementary Fig. 6A). By contrast, DHRS9 downregulation was not consistently observed (data not shown).

Contribution of PIP5K1C to crizotinib resistance in ALK( + ) ALCL was explored by CRISPR interference (CRISPRi) technology. COSTR200 cell line were transduced with dCas9-KRAB-MeCP2 fusion proteins using guide RNAs targeting the PIP5K1C promoter to achieve transcriptional silencing. As shown in Supplementary Fig. 6B, gRNA-mediated silencing of PIP5K1C resulted in a significant reduction in PIP5K1C protein expression. Compared with control cells, PIP5K1C-silenced cells exhibited a slight decreased viability in vitro, when grown in the presence of 1 µM Crizotinib (Supplementary Fig. 6C).

These results indicate that regulation of PIP5K1C by circZBTB46 participates in promoting resistance to ALK inhibition in ALK( + ) ALCL.

### CircZBTB46 regulates PIP5K1C by sponging miR25-3p in ALK( + ) ALCL cells

After RNA pull-down was performed using a back-splice junction-specific probe in ALK( + ) ALCL cells, RT‒qPCR (Supplementary Fig. 7A) confirmed a successful enrichment of circZBTB46. Mass spectrometry analysis of associated proteins did not reveal any specific interactors (data not shown), whereas small RNA sequencing identified four miRNAs (cutoff of |log2Fold Change | ≥ 2 and a *P* value < 0.05), namely, hsa-miR-22-3p, hsa-miR-222-5p, hsa-miR-10a-5p, and hsa-miR-25-3p, that were significantly enriched in the circZBTB46 pull-down compared with control conditions (Fig. 6E, Supplementary Tables S5 and S6). With the exception of hsa_miR_222-5p, the other three miRNAs were expressed in ALK( + ) ALCL patients (Supplementary Fig. 7B), hsa-miR-25-3p being notably overexpressed compared with RLN ([34]. Using miRNA–mRNA interaction prediction algorithms (miRDB, TargetScanHuman and miRTargetLink 2.0), PIP5K1C was identified as a putative target of miR-25-3p (Supplementary Fig. 7C, D). Consistently, transfection of a miR-25-3p mimic in crizotinib-sensitive (PIO) and crizotinib-resistant (COSTR200) cells, as validated by RT‒qPCR analysis (Fig. 6F), led to a reduction in PIP5K1C protein level (Fig. 6G). Conversely, transfection of a miR-25-3p inhibitor significantly increased *PIP5K1C* mRNA and protein levels as observed in in COSTR200 cells, further validating this regulatory pathway (Supplementary Fig. 7E, F). Together, these data indicate that circZBTB46 modulates PIP5K1C levels via miR-25-3p sequestration, uncovering a novel competing endogenous RNA axis in ALK( + ) ALCL.

### Targeting circZBTB46 in ALK( + ) ALCL cells reduces tumor growth in vivo

In vitro experiments revealed that circZBTB46 is involved in crizotinib resistance in ALK( + ) ALCL cells. To evaluate its potential as therapeutic target, the effect of circZBTB46 targeting on tumor growth was analyzed after subcutaneous injection in NSG mice.

**Fig. 7:**
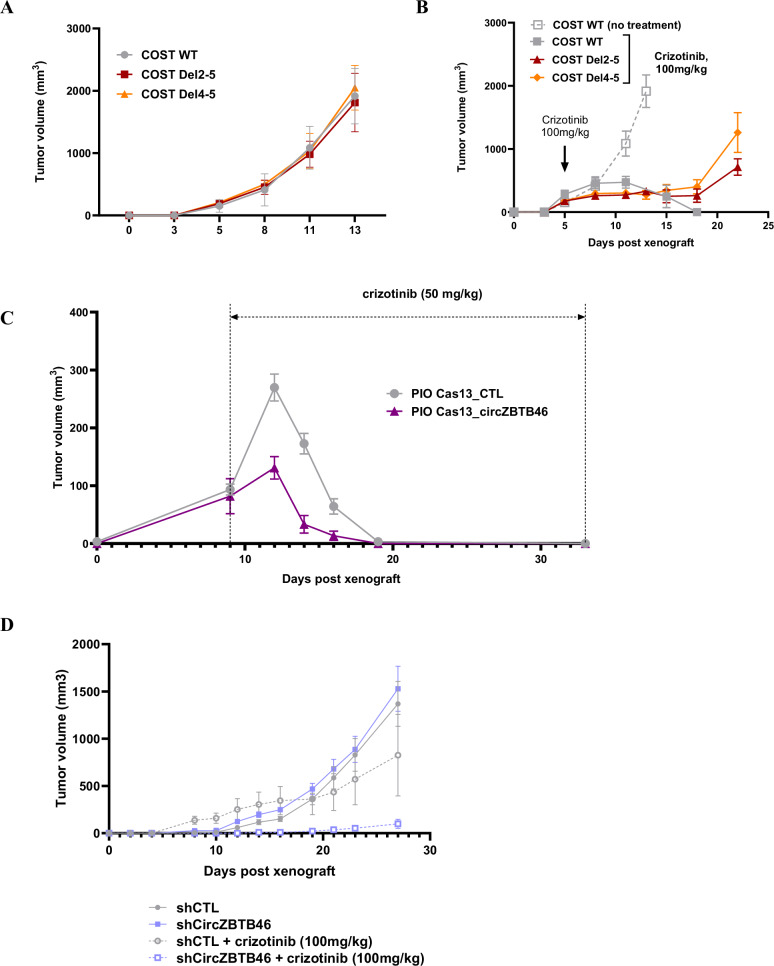
circZBTB46 modulates crizotinib resistance in vivo. Tumor growth in NSG mice (*n* = 8) injected subcutaneously (**A-B**) with COST cells (**A**) untreated or (**B**) treated with crizotinib (5 mg/kg/day); **C**) with PIO cells expressing Cas13 with either a nontargeting control gRNA (Cas13_CTL) or a gRNA targeting circZBTB46 (Cas13_circZBTB46) and (**D**) with COSTR200 cells transduced with or without circZBTB46 shRNA with or without crizotinib treatment (100 mg/kg/day). Parental cells were included as a control. Experiments were performed in triplicate. Tumor volume was monitored over time via calipers and is expressed as the mean ± SEM.

First, CRIPSR/Cas9 crizotinib-sensitive models were engrafted. Del2-5 and Del4-5 clones formed tumors at comparable rates (Fig. 7A). Daily administration of crizotinib, started five days after transplantation, initially reduced the tumor volume in all groups. However, by day 15, all tumors derived from ZBTB46 RNA-depleted clones resumed growth, whereas those derived from parental (WT) cells continued to regress (Fig. 7B). Notably, tumors derived from Del2-5 clones lacking circZBTB46 exhibited slower growth than those derived from circZBTB46(+) clones (Del4-5), corroborating in vitro findings. Next, Cas13-modified crizotinib-sensitive PIO cells were xenografted into NSG mice and treated daily with crizotinib since nine days after injection. Tumor growth was delayed in mice bearing circZBTB46-deficient tumors (Fig. 7C). As PIP5K1C was identified as a downstream target of circZBTB46, PIP5K1C CRISPRi models were also injected in NSG mice. This resulted in a slight reduction of tumor growth upon crizotinib administration (Supplementary Fig. 8A). This highlights the role of PIP5K1C as a mediator of the ITK resistance phenotype. Finally, the crizotinib resistant shCircZBTB46 models were injected to determine whether targeting circZBTB46 in crizotinib-resistant cells could restore drug sensitivity. We confirmed that PIP5K1C protein expression was efficiently reduced in tumors after shCircZBTB46 (Supplementary Fig. 8B). Mice were daily treated with crizotinib or vehicle. In the absence of TKI treatment, tumor growth did not differ between the groups. However, upon crizotinib treatment, tumors derived from shCircZBTB46 cells exhibited significantly reduced growth (Fig. 7D). All these results support the contribution of circZBTB46 to crizotinib resistance in ALK( + ) ALCL and identify circZBTB46 as an interesting therapeutic target of TKI-resistance to in ALK( + ) ALCL disease.

## Discussion

This study identifies the circular RNA, hsa_circ_0002805 (circZBTB46), as a key driver of crizotinib resistance in ALK-positive anaplastic large cell lymphoma (ALK( + ) ALCL). This circRNA, derived from exons 2 and 3 of the ZBTB46 gene, was found to be significantly and specifically upregulated in this lymphoma type. The upregulation is driven transcriptionally by the STAT3 pathway, which is activated by the oncogenic NPM1::ALK fusion protein. Using a combination of CRISPR/Cas and RNAi technologies, we functionally dissected the role of circZBTB46, finding that it, unlike its linear protein-coding counterpart, promotes resistance to crizotinib, an ALK inhibitor. This was demonstrated in both in vitro and in vivo models. Depleting circZBTB46 in resistant cells restored their sensitivity to crizotinib, highlighting its potential as a therapeutic target. Transcriptomic analysis revealed that PIP5K1C is a downstream effector of the circZBTB46-mediated resistance. Mechanistically, circZBTB46 acts as a molecular sponge for miR-25-3p, a microRNA that normally suppresses PIP5K1C expression. By sequestering miR-25-3p, circZBTB46 de-represses PIP5K1C, promoting survival signals and contributing to drug resistance. This proposed circZBTB46–miR-25-3p–PIP5K1C axis is a novel resistance mechanism in hematologic malignancies.

ZBTB46 is a transcription factor normally restricted to dendritic cell precursors, where it maintains their immature state [21, 34–36]. It is also found in non-proliferative endothelial cells, where it inhibits cell proliferation [27]. Emerging evidence, however, points to a context-dependent role for ZBTB46 in cancer [37–39]. In acute myeloid leukemia (AML), elevated ZBTB46 is associated with poor outcomes [40], and despite not being essential for normal hematopoiesis, it is crucial for the survival and proliferation of AML cells [41].

Similarly, circZBTB46 exhibits context-dependent functions across different diseases. In atherosclerosis, circZBTB46 promotes disease progression by activating the AKT/mTOR pathway [42]. In this context, circZBTB46 depletion induces apoptosis and impairs cell migration, highlighting its importance in vascular pathology. In contrast, this study found no effect on AKT/mTOR signaling in ALK( + ) ALCL cells upon circZBTB46 silencing (data not shown).

In AML, circZBTB46 has been identified as an oncogenic effector that promotes cell survival. It acts as a competing endogenous RNA, sponging both hsa-miR-326 and hsa-miR-671-5p. This activity increases the expression of stearoyl-CoA desaturase 1 (SCD1), an enzyme that protects cells from lipid peroxidation and ferroptotic cell death. These findings establish circZBTB46 as a novel oncogenic effector in AML and reveal an unrecognized *ZBTB46*-derived noncoding RNA axis that governs cell fate through ferroptosis-related mechanisms [40]. Notably, both *ZBTB46* linear RNA and circZBTB46 were found to be expressed at markedly higher levels in ALK( + ) ALCL than in AML. This was demonstrated by comparative transcriptomic analyses using a k-mer-based approach [43, 44] applied to in-house RNA-seq data from primary biopsy samples, including ALK( + ) ALCL and AML samples (IUCT-AML cohort) (Supplementary Fig. 9) [45, 46]. RLN and established cell lines (AML and ALK( + ) ALCL), including *NPM1::ALK*–transformed immortalized T-cell models [19, 29], and healthy T lymphocytes confirmed these data. This unexpected expression pattern underscores the context-dependent transcriptional regulation and functional plasticity of the *ZBTB46* locus across distinct hematological malignancies. In fact, this study expands knowledge on the pathophysiological oncogenic potential of ALK( + ) ALCL by demonstrating the role of circZBTB46 in promoting resistance to crizotinib. It was shown here that circZBTB46 is transcriptionally upregulated via the NPM1::ALK/STAT3 signaling axis, thereby linking oncogenic kinase activity to circular RNA-mediated drug resistance. These findings suggest that circZBTB46 acts as a context-specific regulator of therapy resistance by engaging survival pathways, making it a potential therapeutic target in ALK-driven hematological malignancies. In this context, PIP5K1C appears as a downstream target of circZBTB46. miR-25-3p was identified as a candidate intermediary. miR-25-3p has previously been shown to target PIP5K1C in prostate cancer [47]. These findings support a model in which circZBTB46 may act as a molecular sponge for miR-25-3p, thereby derepressing PIP5K1C expression and promoting survival signaling. Thus, circZBTB46 might contribute to crizotinib resistance in ALK( + ) ALCL through a miR-25-3p–PIP5K1C axis, a mechanism not previously appreciated in hematologic malignancies.

The miR-25–PIP5K1C interaction, which has already been validated in resistant prostate cancer [47], is also relevant here in ALK-positive ALCL, where PIP5K1C plays a role in resistance to ALK-targeted therapy. This lipid kinase catalyzes the phosphorylation of phosphatidylinositol 4-phosphate (PI4P) to produce phosphatidylinositol 4,5-bisphosphate (PI(4,5)P₂), which plays a key role in remodeling the actin cytoskeleton, intracellular trafficking and cell migration [48]. PIP5K1C phosphorylation at serine 448 has been suggested to be a potential biomarker for breast cancer progression, particularly in invasive breast cancer [49]. Compared with their sensitive counterparts, melanoma cells that are resistant to the PIKFYVE inhibitor WX8 exhibit elevated expression of PIP5K1C, and this resistance can be reversed by either siRNA-mediated knockdown of PIP5K1C or pharmacological inhibition of its kinase activity [50]. These findings suggest that PIP5K1C could mediate therapeutic resistance in multiple cancer types including hematological neoplasms. Therefore, focusing on PIP5K1C in crizotinib-resistant ALCL cells could be an effective way to overcome resistance.

## Methods

### Cell line culture

The ALCL cell lines KARPAS-299, SU-DHL-1 SUPM2, PIO and COST, which carry the t(2;5)(p23;q35) translocation, were obtained from the DSMZ (German Collection of Microorganisms and Cell Culture, Leibnitz, Germany) or established locally [33]. The MTK PDX-derived cell line was provided by J. Mathews and S. Turner [32], and the crizotinib-resistant KARPAS-299-CR06 cell line was provided by C. Gambacorti-Passerini [51]. COST cells were exposed to increasing concentrations of crizotinib, ranging from 25 nM to 200 nM. At each step, the dose was increased only when treated cells achieved a proliferation rate comparable to that of untreated wild-type (WT) cells. All NPM::ALK( + ) ALCL cell lines and PDXs were cultured in RPMI-1640 medium (Invitrogen, Waltham, MA #61870044) supplemented with 20% FBS (Pan Biotech, Aidenbach, Germany #500105M1M). For crizotinib-resistant cell lines, crizotinib was added to the medium twice a week at a concentration of 200 nM for COSTR200 and 600 nM for KARPAS-299-CR06. The cancer cell lines employed in this study had been genotyped in advance to verify the expected mutations. Cells were cryopreserved and maintained at early passages. Following thawing, each line was passaged at least once, and Mycoplasma contamination was assessed (Lonza, LT07-710) before implantation into mice.

### Crizotinib, siRNA and mimic treatments

Cells were seeded at 2 × 10^5^ cells/mL and treated at 24 h with different doses of crizotinib (Selleckchem, Houston, TX, PF-02341066 #S1068). Aliquotes of 3.10^6^ cells were transfected with 70 to 100 nmol siRNA/mimic (listed in Supplementary Table S7) using Amaxa4D nucleofector (Primary Cell kit P3, CA-137 program, Lonza Bioscience, Antwerp, Belgium).

### RNA pulldown

iDRIP was performed as previously described [52]. Probe sequences are listed in Supplementary Data Table S7.

### Xenograft tumor assay

Mice were housed under pathogen-free conditions in an animal room at constant temperature (20 °C–22 °C) with a 12 h light/dark cycle and free access to food and water. A total of 3 × 10^6^ cells were injected subcutaneously into one flank of 5-week-old female NSG mice (Janvier Labs, Le Genest Saint Isle, France). Mouse body weight and tumor volumes were measured 3 times a week with calipers via the following formula: length × width^2^× π/6. At the end of the experiment, the mice (8 per group) were humanely sacrificed. For crizotinib-resistant cells, mice were treated with 100 mg/kg/day crizotinib starting on the day following injection. For crizotinib-sensitive cells, mice were treated with 50 mg/kg/day crizotinib, starting when the tumor size reached 300 mm^3^. For animal studies no randomization and no blinding were used. All animal procedures were performed following the principle guidelines of INSERM. Our protocol was approved by the Midi-Pyrénées Ethics Committee on Animal Experimentation and conducted in accordance with institutional guidelines and the Directive 2010/63/EU under protocol number DAP-APAFiS-2021100714596279-, CEEA122 agreement: 2021100714596279_v9). All experiments were conducted in compliance with all relevant guidelines and regulations, including the Animal Research: Reporting of In Vivo Experiments (ARRIVE) guidelines.

### Statistical analyses

Data are expressed as mean ± standard error of the mean (SEM) of at least three independent experiments. Graphs and statistical analyses were performed via GraphPad Prism 10 (GraphPad Software Inc., La Jolla, CA). P values were calculated by using an unpaired two-tailed Student *T* test with Welch correction.

### CircRNA validation by PCR and Sanger sequencing

The validation of circRNA back-splice junctions, circularity, and stability were performed as previously. In brief, PCR amplification was followed by Sanger sequencing to confirm the back splicing junction and the full circRNA sequence. Circularity and exonuclease resistance were assessed via RNase R treatment, whereas transcript stability was evaluated following exposure to actinomycin D. Relative expression levels were determined via RT‒qPCR via the 2-∆∆Ct method.

## Supplementary information

supplementary information

Supplemental_Table S1

Supplemental_Table S2

Supplemental_Table S4

Supplemental_Table S5

Supplemental_Table S6

Supplemental_Tables S3 and S7

## Data Availability

The ribo zero full RNA sequencing, the long-read Oxford Nanopore RNA sequencing and STAT3 ChIP-seq datasets are available in the GEO repository (GSE160123, GSE197872 and GSE117164 respectively). The RNA sequencing after siRNA against circZBTB46 is available in ENA repository (PRJEB109936). The patient samples RNA-sequencing datasets generated are available from the corresponding author on reasonable request.
