# Supplementary material for: CircZBTB46, a promising therapeutic target in crizotinib resistant ALK-positive T lymphomas
Source: Leukemia. 2026 Jun 26;40(8):1735–47. doi: 10.1038/s41375-026-03018-w (PMC13421333; doi:10.1038/s41375-026-03018-w)
Supplement: Supplementary file 1 — supplementary information [file 41375_2026_3018_MOESM1_ESM.pdf]

Supplementary Data

Supplementary methods:

CRISPR model/shRNA model cell lines

Guide RNAs for CRISPR/Cas13 models were cloned and inserted into the pSLQ5465_pHR_hU6-crScaffold_EF1a-PuroR-T2A-BFP backbone (Addgene, Wartertown, MA, #155307) via BbsI digestion. Guide RNAs for CRISPRi were subsequently cloned and inserted into the pKLV2-U6gRNA5(BbsI)-PGKpuro2ABFP-W backbone (Addgene #67974) via BbsI digestion. shRNAs were cloned and inserted into the pLKO.1 puro backbone (Addgene #8453) via AgeI and EcoRI digestion. All sequences are listed in Supplementary Table S7. Lentiviruses were produced in HEK293T cells. The cells were transfected with psPAX2 (Addgene #12260), pMD2. G (Addegene # 12259) and the plasmid of interest using Lipofectamine 2000 and 24 µg total DNA (with a ratio of 1:1:1). Lentiviruses were harvested 48 h after transfection and used directly on ALCL ALK(+) cells. A total of 10^6 cells were transduced with 1 mL of lentiviral supernatant supplemented with 10 µg/mL polybren (Sigma, St Louis, MO, #TR-1003-G). Cells were spun-inoculated for 2 hours at 1000 rpm and 37°C, after which 1 mL of fresh medium was added. For the CRISPR/Cas13 model, cells were first transduced with pXR001:EF1a-CasRx-2A-EGFP lentivirus (Addgene #109049) and then with pSLQ5465_pHR_hU6-crScaffold_EF1a-PuroR-T2A-BFP lentivirus containing guides of interest. The transduced cells were then selected with puromycin (1 µg/mL). For CRISPRi models, cells were first transduced with dCas9-KRAB-MeCP2 lentivirus (Addgene #110821) and then with pKLV2-U6gRNA5(BbsI)-PGKpuro2ABFP-W lentivirus containing guides of interest. The transduced cells were then selected with puromycin (1 µg/mL). For the shRNA model, cells were transduced with pLKO.1 puro lentivirus containing the shRNA sequence of interest.

Western blot analysis

Whole-cell extracts were prepared with protein lysis buffer (50 mM Tris-HCl pH 7.4, 1%Triton X-100, 0.1% SDS, 150 mM NaCl, 1 mM EDTA, and 1 mM DTT), supplemented with « Complete cocktail protease inhibitor tablets » (Roche, Vienna, Austria, #11697498001) and « Halt Protease and Phosphatase Inhibitor Cocktail » (Thermo Fisher Scientific, Waltham, MA #78440). Typically, 30 µg of protein were loaded on 10% acrylamide gel (w/v) Tris-HCl SDS PAGE. After transfer, membranes were stained with anti-Phospho-ALK (Tyr1604 #3341 RRID: AB_331047), anti-ALK (31F12 #3791, RRID: AB_1950402), anti-P-STAT3 (Tyr705 #9131, RRID: AB_331586), anti-STAT3 (#4904, RRID: AB_331269), anti-PIP5K1C (#3296,

RRID:AB_2164719) from Cell Signaling and anti-ZBTB46 (Sigma Aldrich, St Louis, MO, #HPA013997, RRID: AB_1858961, an antibody directed against an epitope encoded by exon 2) from Atlas Antibodies. Anti-Tubulin (Santa Cruz, Dallas, TX, #sc-53029 RRID : AB_793541) Anti-actin (Sigma, #A5316, RRID : AB_476743) and anti-GAPDH (Santa-Cruz, #sc-32233, RRID : AB_627679) antibodies were used as loading controls. Secondary antibodies were HRP-conjugated, and blot developed with ECL prime (BIO-RAD, Hercules, CA, # 1705060).

Immunohistochemistry

ALK, CD30, CD68, CD163 and ZBTB46 protein expression was assessed by immunohistochemistry on formalin-fixed, paraffin-embedded (FFPE) tissue sections. The protein expressions were detected by immunohistochemistry via a polyclonal rabbit anti-human ALK antibody (clone SP8; 1:100 dilution; Lab Vision Corporation, Fremont, CA), a monoclonal mouse anti-human CD30 antibody (clone JCM182; ready-to-use; Leica Biosystems, Buffalo Grove, IL, USA), a monoclonal mouse anti-human CD68 antibody (clone PG-M1; 1:100 dilution; Dako, Les Ulis, France), a monoclonal mouse anti-human CD163 antibody (clone MRQ-26, ready-to-use, Roche Cell Marque, Rocklin, CA, USA) and a rabbit polyclonal antibody anti-human ZBTB46 antibody (clone NBP1-88506, 1:100 dilution; Novus Biologicals, Centennial, CO, USA). Signal visualization was achieved via the use of a streptavidin–biotin–peroxidase complex Vector Laboratories, Burlingame, CA). Hematoxylin was used as a counterstain to highlight the nuclei and overall tissue architecture.

Immunofluorescence

ALCL cells (150,000 per well) were seeded overnight in FCS-free medium on Poly-L-Lysine–coated 8-well chamber slides (Ibidi, Gräfelfing, Germany #80827). Cells were fixed with 4% paraformaldehyde (Thermo Fisher, #043368.9M), permeabilized with 0.5% Triton X-100, then washed with 0.1% Tween-20 in PBS. Non-specific binding was blocked using 10% FCS. Cells were incubated with rabbit anti-ZBTB46 antibody (1:100; Atlas, Stockholm, Sweden, #HPA103997, RRID:AB_1858961) in 2% FCS/PBS, followed by a 2-h incubation at RT with Alexa Fluor 568-conjugated goat anti-rabbit secondary antibody (1:2000; Thermo Fisher, #A11011, RRID:AB_143157) in 2% FCS/PBS. After washing, slides were mounted with DAPI-containing medium (Thermo Fisher, #S36920). Imaging was performed using a Zeiss LSM 880 confocal microscope in Airyscan mode with Zen Black software.

Apoptosis assay

Apoptosis was detected via a Pacific Blue™ Annexin V kit (BioLegend #640918) or an APC/Fire™ 750 Annexin V (BioLegend, San Diego, CA #640953) following the manufacturer's instructions. Flow cytometry analysis was performed on a MACSQuant® (Miltenyi Biotec, Paris, France) Analyzer 10 flow cytometer, and the data were analyzed with FlowJo software(BD Biosciences, San Jose, CA).

Cell cycle analysis

Cell cycle analysis was performed via an EdU Click-iT kit (Invitrogen, Waltham, MA #C10337) following the manufacturer's instructions.

RNA extraction, reverse transcription and quantitative PCR

Total RNA was extracted from 5 to $10x10^6$ cells via TRIzol reagent (Invitrogen # 15596018) followed by a Direct-zol™ RNA Miniprep Plus Kit (Zymo, Orange, CA #R2072) following the manufacturer's instructions. Typically, 1 µg of total RNA was reverse transcribed via the ProtoScript® II First Strand cDNA Synthesis Kit (NEB, Ipswitch, MA, #E6560L) following manufacturer instructions. cDNA was next diluted with 5 in water, and 2 µL were used for quantitative PCR analysis. qPCR was performed via the Master Mix Select SYBR™ (Applied Biosystems, Waltham, MA, #4472908) in a 10 µL total reaction. The PCR program was as follows: 50°C for 2 min, 95°C for 2 min, and 40 cycles of 95°C for 15 s and 60°C for 1 min. All primer sequences are listed in Supplementary Table S7.

MicroRNA detection

Retro transcription was performed from 10 µg total RNA using miRCURY LNA RT Kit (Qiagen, Les Ulis, France # 339340), following manufacturer's instructions. qPCR quantification was performed using miRCURY LNA SYBR Green PCR Kit (Qiagen #339345) following manufacturer's instructions. SNORD44 RNA was used as housekeeping gene. Probes were listed in Supplementary Table S7.

Chromatin immunoprecipitation

Fifteen million cells were fixed in 2% paraformaldehyde. Chromatin immunoprecipitation was then performed via a ChIP-IT® Express Kit (Active Motif, Shangai, China, #53008) according to manufacturer instructions. Sonication was performed on VibraCell (Sonics & Materials, Newton, CT, #75186) 10 times for 20s at a power of 25s (with a 20s pause in between). Immunoprecipitation was performed using an anti-STAT3 antibody (#12640S, RRID:

AB_2629499) and an anti- rabbit IgG antibody (#2729, RRID: AB_1031062) from Cell Signaling Technology (Danvers, MA).

Protein extraction from tumors

Small tumor fragment (~20 mg) was lysed in protein lysis buffer (see Western blot section for buffer composition) using Precellys system with steal beads (#P000925-LYSK0-A). Program used : 20s on, 20s off, 20s on.

RNA dataset generation and analysis

Microarray data (.CEL files) for patients with ALCL, AITL and PTCL-NOS were retrieved from the translational T-cell lymphoma research consortium (TENOMIC) of the LYmphoma Study Association (LYSA) and PAIR lymphoma project, previously available (1, 2). Raw data were processed with the *affy* R package and the *rma* function to transform probe intensities into normalized expression values by robust multi-array average (RMA) (Irizarry et al Biostatistics 2003). To get by-gene summarized expression values, the collapse microarray tool was used (https://sites.google.com/site/fredsoftwares/products/collapse-microarray?authuser=0) with the provided Affymetrix U133+2 table corresponding to the chip reference.

The Ribo zero full RNA sequencing for 39 ALCL patient samples and 9 RLN samples was previously published (GSE160123, (1)). For all other samples, the same library preparation was performed. Pooled library prep samples were subsequently sequenced on a NovaSeq 6000 Illumina (San Diego, CA), corresponding to 1x30 million 100-base reads per sample after demultiplexing. For ZBTB46 mRNA expression analysis shown in Fig. 1E, reads pseudoalignment was performed with Kallisto (3) (version 0.46.1) using the v108 Ensembl transcriptome, followed by *tximport* (4) for computing gene-level TPM values, normalized by gene length and library size (lengthScaledTPM). De novo identification of circRNAs was performed according to a standard protocol, using the CIRI2 algorithm with the human reference genome GRCh38 (5). The cutadapt-3.4 software (6) was used to trim Illumina adaptors and discard reads that were less than 30 nt in both ends. The bwa-0.7.17 (7) software was used to align reads to the GRCh38 reference genome in local mode (parameter used: -T 30). After alignment, the CIRI_v2.0.6 software (8, 9) was used to predict circRNA candidates. The number of reads aligning to the back-splicing junctions was used as the expression level of each circRNA. Differential expression analysis was performed using the DESeq2 tool (10).

For all other samples, the reads were aligned with hisat2 (11) on the hg38 human genome and expression was quantified via featureCounts (12). Differential expression analysis was performed via the DESeq2 tool (10).

SmallRNA library preparation was performed according to manufacturer recommendations (QIAseq miRNA Library Kit from Qiagen, LesUlis, France). The final pooled library preparation samples were sequenced on Novaseq 6000 Illumina corresponding to 1x30M 100bases reads per sample after demultiplexing. The quantification of miRNAs was carried out according to recommendations by Potla et al (13).

Supplementary Figures

Supplementary Figure 1. Expression of *ZBTB46* in ALK(+) and ALK lymphoma

A

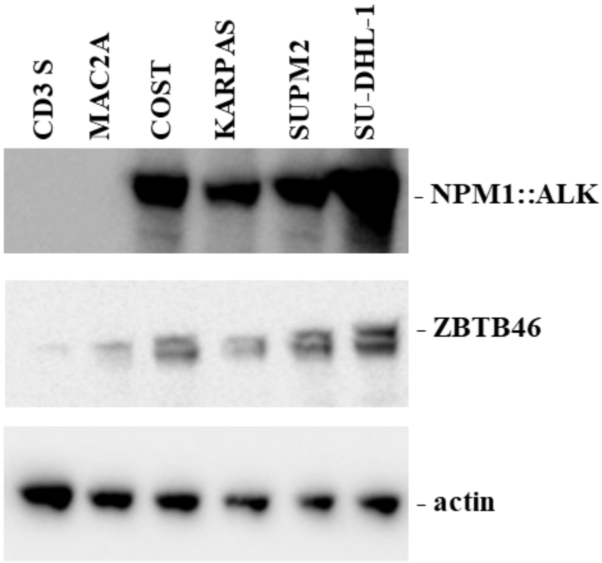

B

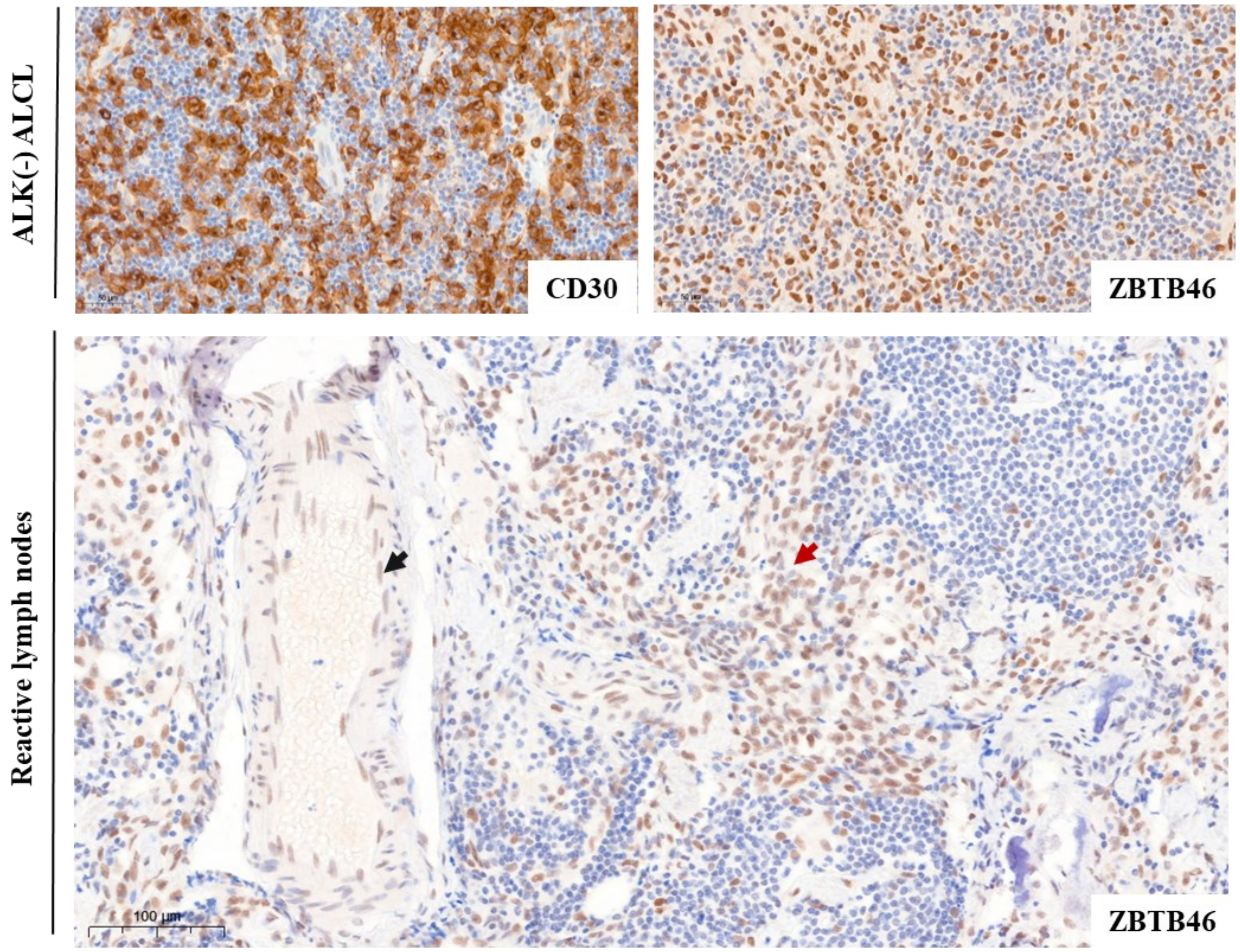

endothelial cells, sinusoidal macrophage-like cells

(A) Expression of ZBTB46 protein in ALK(+) and ALK lymphoma cells and controls. ZBTB46 and ALK protein expression in ALK(+) ALCL cell lines (KARPAS-299, SU-DHL-1, SUPM2 and COST), MAC2A ALK(-) ALCL cell line and CD3(+) stimulated lymphocytes (CD3 S) was assessed via Western blot analysis. Actin was used as a loading control. **(B) Expression of ZBTB46 protein in ALK(-) primary biopsies and healthy tissues.** Representative immunohistochemical image of an ALK(-) ALCL primary tumor showing CD30 and ZBTB46 expression in tumoral cells (original magnification, ×15). In reactive lymph nodes, CD163 and ZBTB46 are detected in endothelial cells (black arrow) and sinusoidal macrophage-like cells (red arrow) (original magnification, ×22). Cell nuclei were counterstained with hematoxylin (blue).

Supplementary Figure 2. Regulation of *ZBTB46* expression by NPM1::ALK and STAT3 signaling.

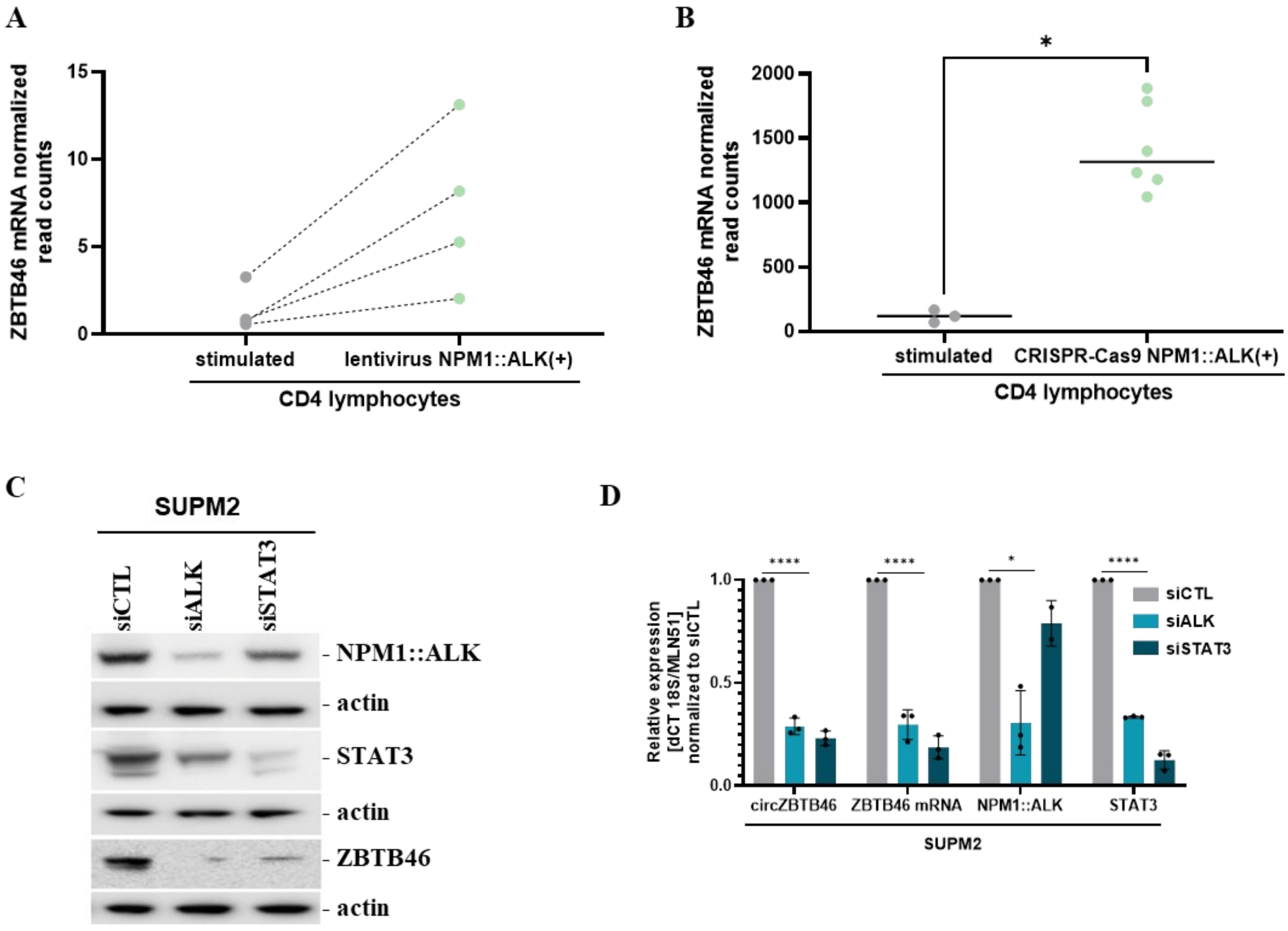

(**A**) Relative expression of *ZBTB46* mRNA, as determined by RNA-Seq, in primary human CD4 T lymphocytes transduced with a lentiviral vector encoding NPM1::ALK (2) and **(B)** *ZBTB46* mRNA expression in human T cells engineered to carry the canonical t(2;5)(p23;q35) translocation by CRISPR/Cas9 genome editing **(5)**. (**C**) Western blot and (**D**) RT–qPCR analysis of ZBTB46, ALK, and STAT3 expression SUP-M2 cells transfected for 48 h with control siRNA (siCTL), ALK-targeting siRNA (siALK), or STAT3-targeting siRNA (siSTAT3). GAPDH or actin were used as a loading controls. MLN51 served as an internal control for RT–qPCR. mRNA expression values are shown as 2-ΔΔCt relative ratios. Experiments were performed at least in triplicate. Statistical significance was assessed via an unpaired two-tailed Student t test with Welch correction: $P < 0.05$ (*); $P < 0.0001$ (****). Data are expressed as means ± SEM.

Supplementary Figure 3. Regulation of *ZBTB46* expression by direct binding of STAT3

A

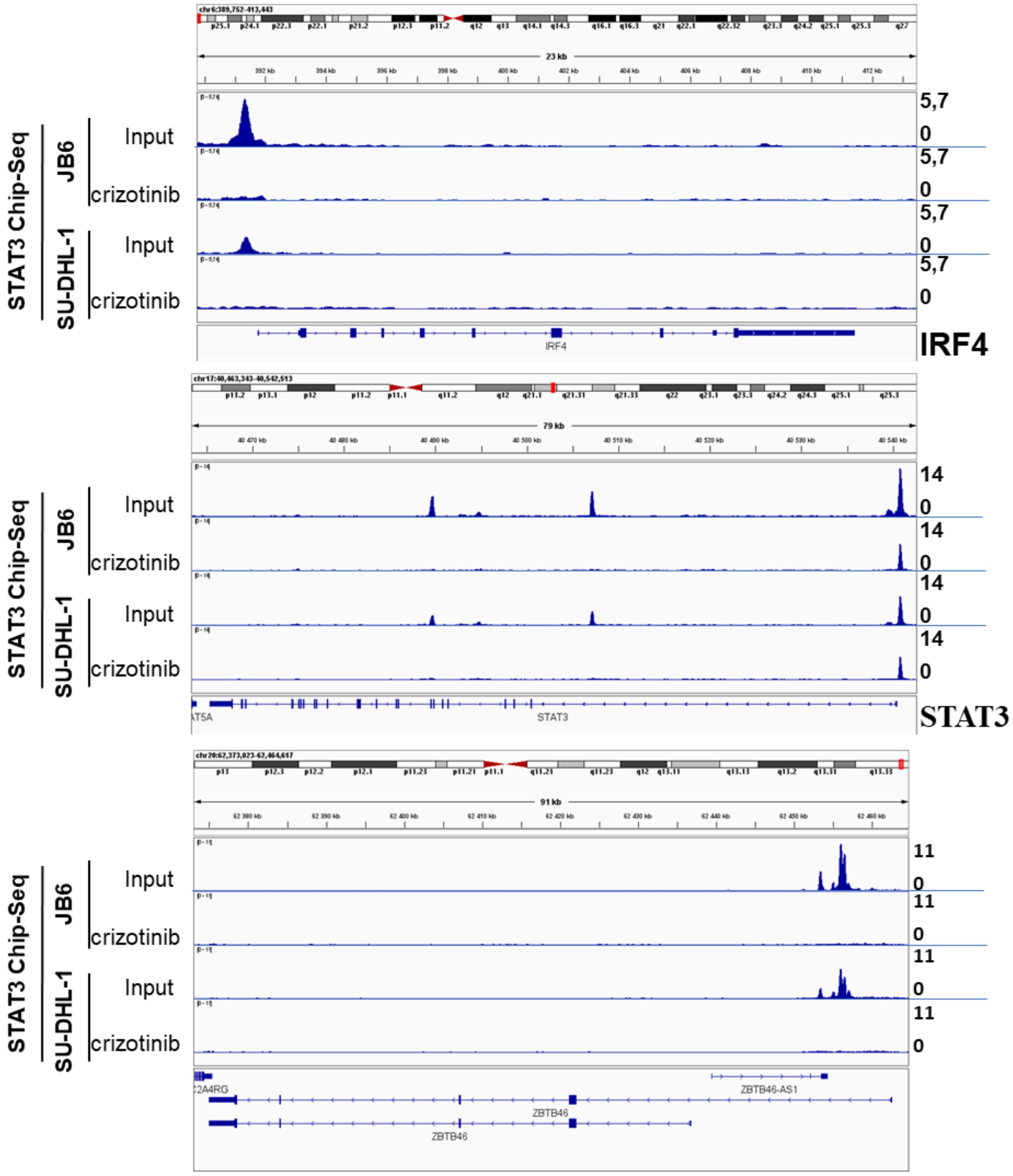

B

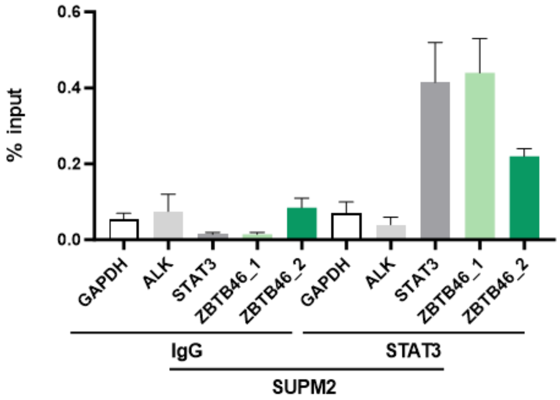

(A) STAT3 chromatin enrichment at the *IRF4*, *STAT3* and *ZBTB46* loci in JB6 and SUDHL1 ALCL cell lines as assessed by *STAT3* ChIP-seq (GSE117164; (14)). Scale for each lane is indicated on the right side. (**B**) ChIP–qPCR analysis using two primer pairs (ZBTB46_1 and ZBTB46_2) showing STAT3 enrichment at the *ZBTB46* promoter in SUP-M2 cells, represented as % input. STAT3 was a positive control and ALK and GAPDH were used as negative controls. IgG was used as a nonspecific binding control.

Supplementary Figure 4. Characterization of *ZBTB46* CRISPR/Cas9 knockout models.

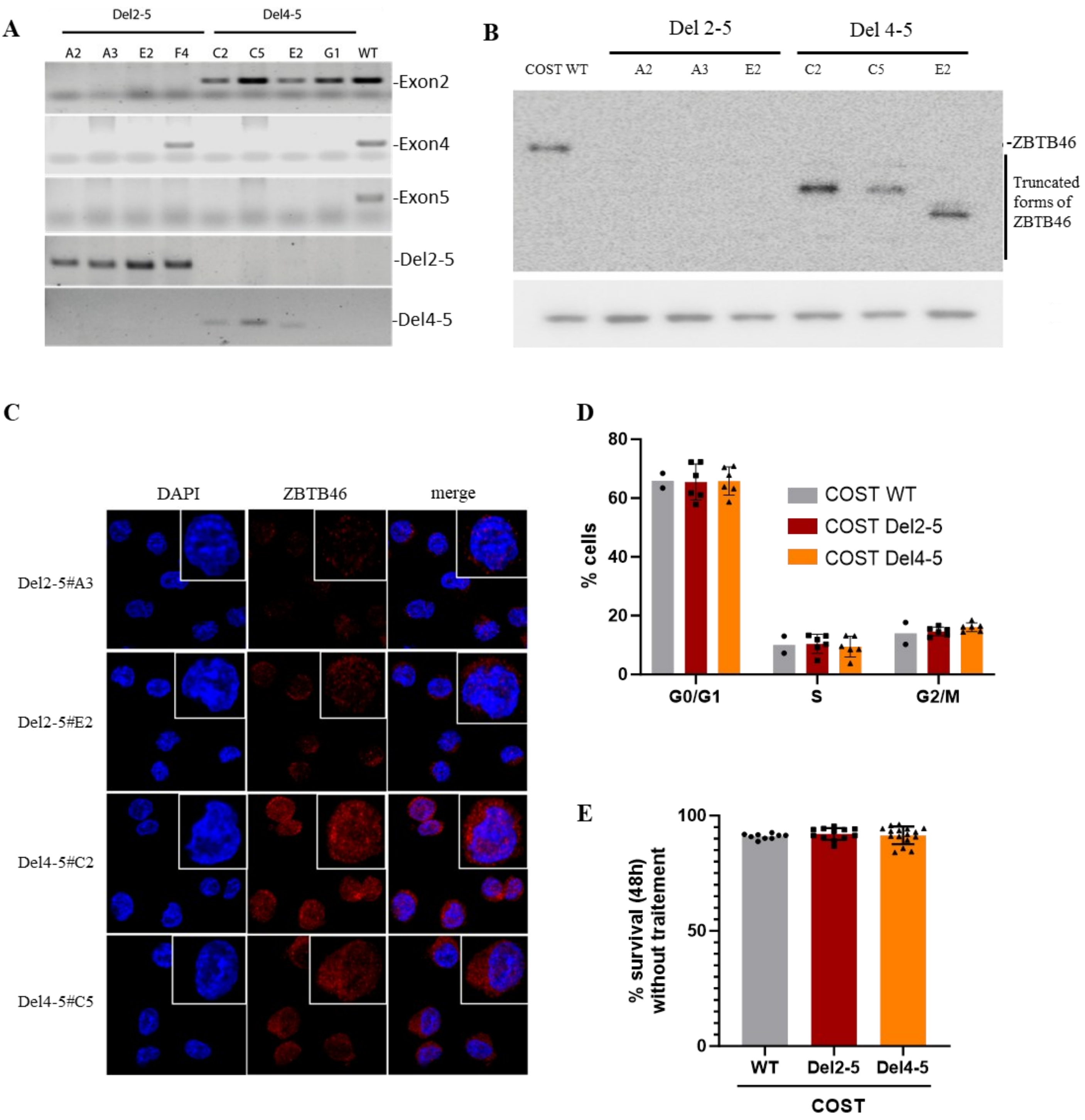

(A) PCR-based detection of *ZBTB46* exon 2-5 (Del2-5) and exon 4-5 (Del4-5) deletions at the DNA level. Amplification of wild-type (WT) exons 2, 4 and 5 was used as a control. **(B)** ZBTB46 protein expression in COST wild-type (WT), Del2-5 and Del4-5 clones as assessed by Western blot using an antibody directed against an epitope encoded by exon 2. Actin was used as a loading control. **(C)** Immunofluorescence staining of the ZBTB46 protein in COST WT, Del4-5 and Del2-5 clones. Original magnification, 63x **(D)** Cell cycle distribution determined by EdU incorporation and flow cytometry analysis of COST WT, Del2-5 and Del4-5 clones.

Supplementary Figure 5. ZBTB46 protein expression after circZBTB46 was targeted via the Cas13 system.

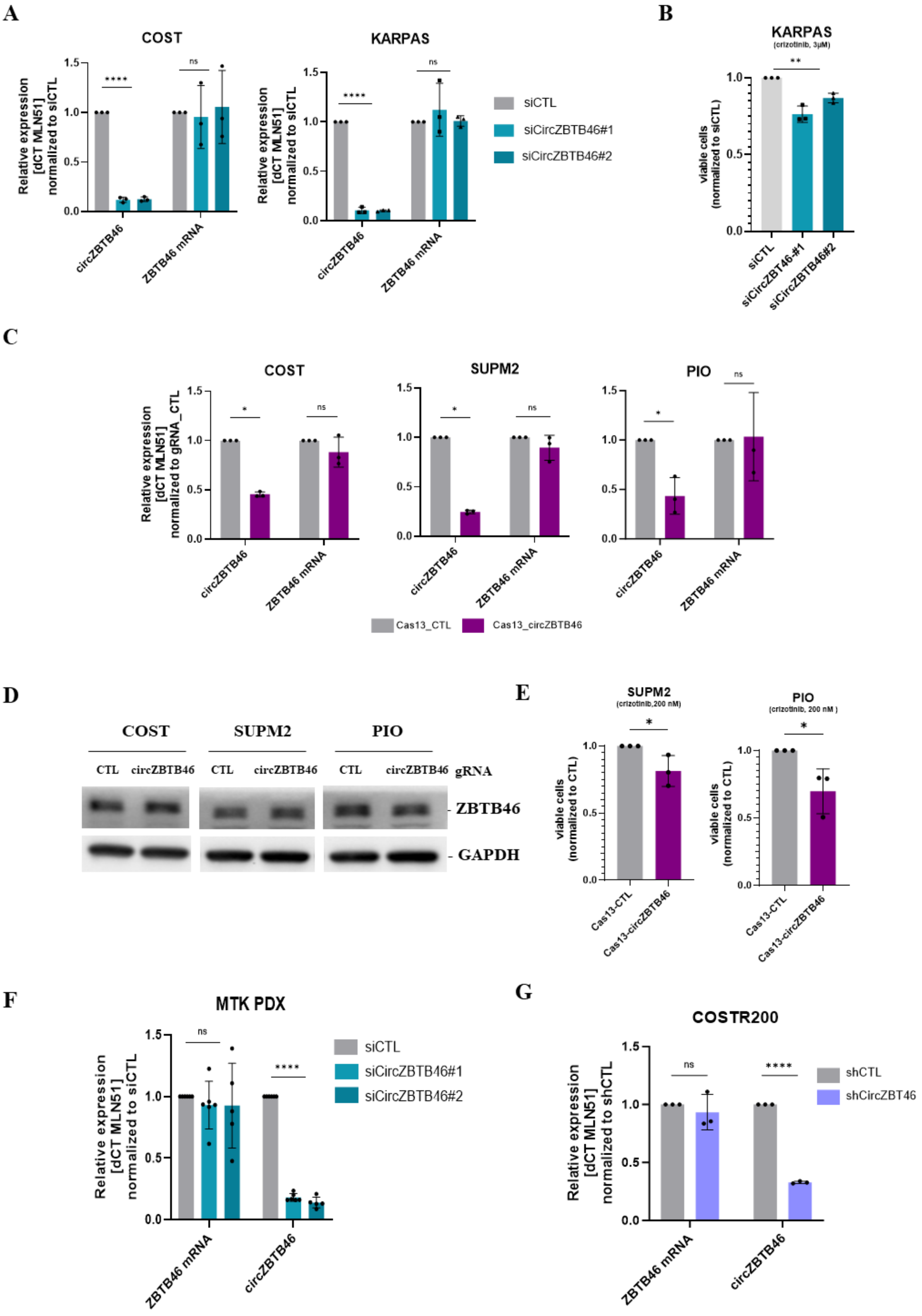

(A) Relative expression of circZBTB46 and *ZBTB46* mRNAs in COST and KARPAS-299 cells transfected for 48 h with control siRNA (siCTL) or two different circZBTB46-targeting siRNAs (siCircZBTB46#1 and #2), as measured by RT–qPCR. **(B)** Viability (Annexin V-Pacific Blue/PI flow cytometry) of KARPAS-299 cells transfected with siCircZBTB46#1 or #2 and subsequently treated with crizotinib (3 µM, 48 h). **(C)** Relative expression of circZBTB46 and *ZBTB46* mRNAs in COST, SUPM2 and PIO cells transduced with Cas13 and either control gRNA (CTL) or gRNA targeting circZBTB46, as assessed by RT–qPCR. **(D)** ZBTB46 protein levels in COST, SUPM2 and PIO ALK(+) ALCL cell lines transduced with Cas13 and either control gRNA (CTL) or gRNA targeting circZBTB46, as assessed by Western blot analysis. GAPDH was used as a loading control. **(E)** Viability (Annexin V-Pacific Blue/PI flow cytometry) of SUPM2 and PIO cells transduced as described in **(C)** and treated with crizotinib (200 nM) for 7 days. (**F**) Relative expression of circZBTB46 and *ZBTB46* mRNAs in crizotinib-resistant cells established from MTK PDX-derived cells transfected for 48 h with control siRNA (siCTL) or two different siRNAs targeting circZBTB46 (siCircZBTB46#1 and #2), as measured by RT–qPCR. (**G**) Relative expression of circZBTB46 and ZBTB46 mRNAs in COST crizotinib-resistant cells (COSTR200) after lentiviral transduction with circZBTB46-targeting shRNAs, as measured by RT–qPCR. GAPDH was used as a loading controls. MLN51 served as an internal control for RT–qPCR. mRNA expression values are shown as 2-ΔΔCt relative ratios. Experiments were performed at least in triplicate. Statistical significance was assessed via an unpaired two-tailed Student t test with Welch correction: $P < 0.05$ (*); $P < 0.01$ (**); $P < 0.0001$ (****); ns = not significant. Data are expressed as means ± SEM.

Supplementary Figure 6. circZBTB46 modulates PIP5K1C expression

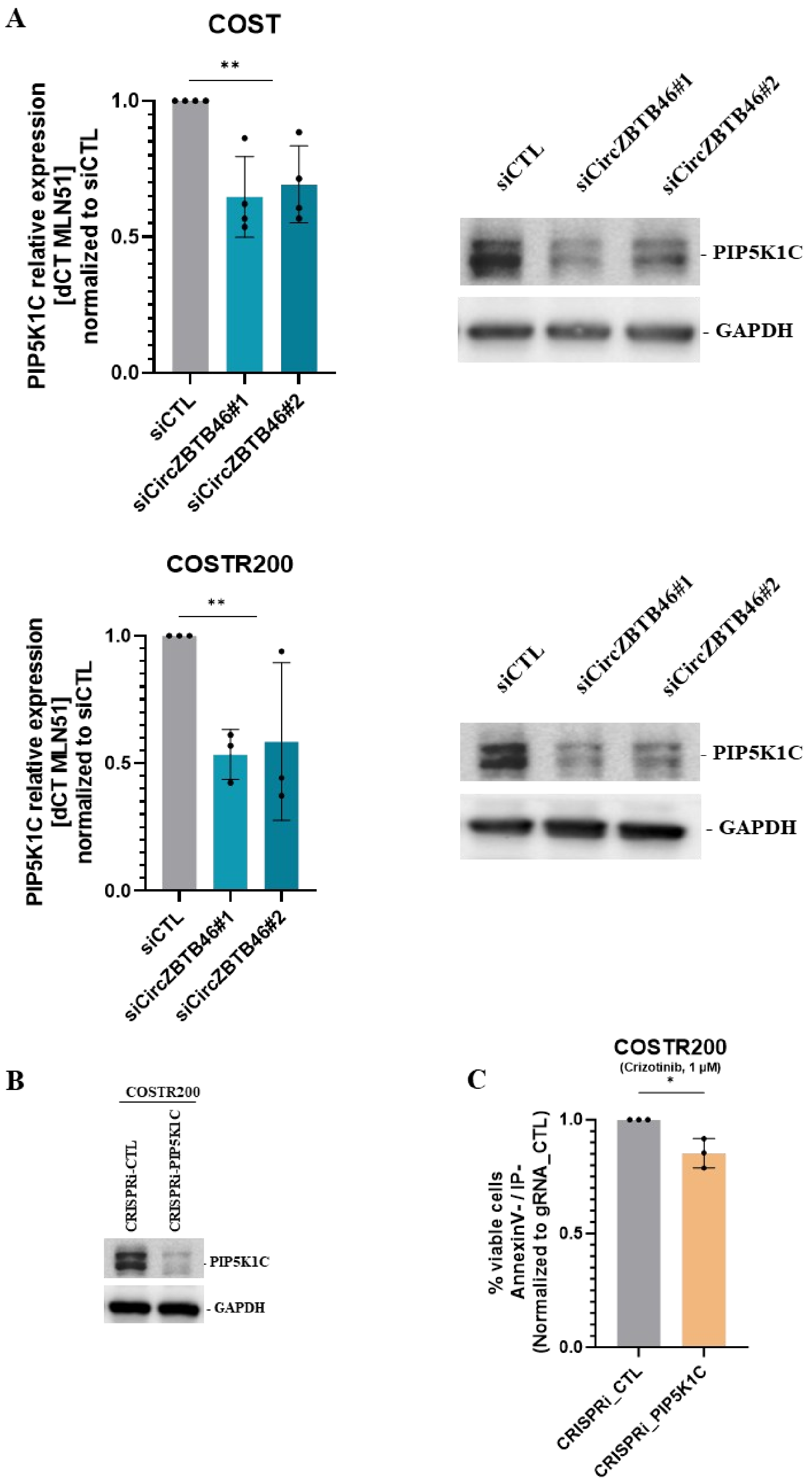

(A) Expression of PIP5K1C at the mRNA (left panel, RT–qPCR) and protein (right panel, Western blot) levels in COST and COSTR200 ALK(+) ALCL cells after circZBTB46 siRNA transfection (48 h). **(B)** PIP5K1C protein levels detected by Western blot in stable COSTR200 cells expressing CRISPRi constructs with control gRNA (CTL) or gRNA targeting the PIP5K1C promoter. **(C)** Cell viability of crizotinib-resistant COSTR200 cells transduced with CRISPRi/gRNA targeting PIP5K1C, followed by crizotinib treatment (1,000 nM, 48 h), as measured by Annexin V/PI staining (flow cytometry). MLN51 served as an internal control for RT–qPCR. mRNA expression values are shown as 2-ΔΔCt relative ratios. Experiments were performed in triplicate. Data are presented as mean ± SEM. Statistical significance was determined using an unpaired two-tailed Student's t test with Welch's correction**:** $P < 0.05$ (*), $P < 0.001$ (**).

Supplementary Figure 7. MiR-25-3p regulates PIP5K1C expression in crizotinib-resistant ALK(+) ALCL cells.

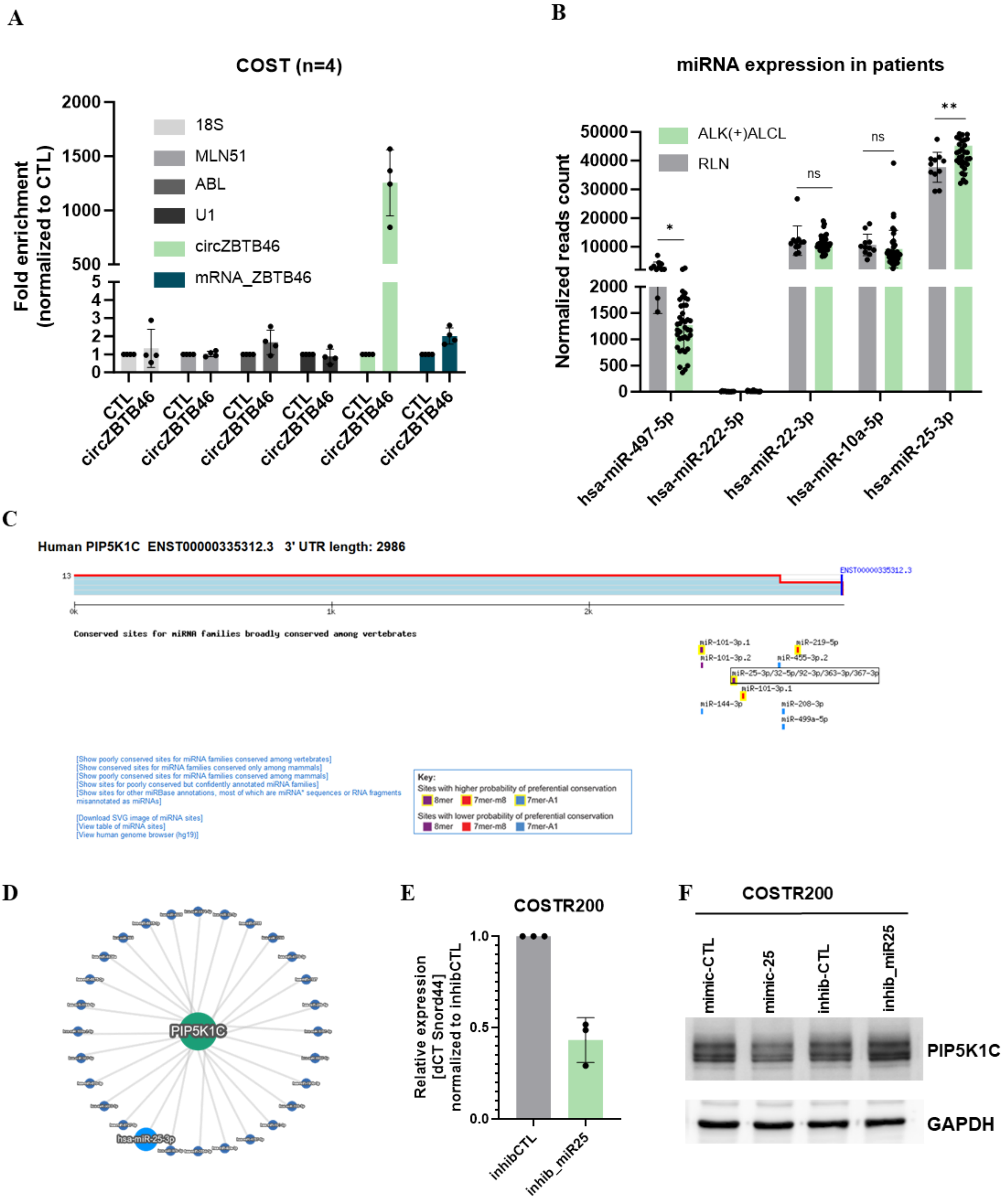

(A) RNA enrichment after RNA pull-down using biotinylated probes targeting circZBTB46 or control probes**. (B)** Relative expression levels of microRNAs (miR_497-5p, miR_222-5p, miR_22-3p, miR_10a-5p, and miR_25-3p) in ALK(+) ALCL primary samples versus reactive lymph nodes (RLNs), as assessed by small RNA sequencing. **(C-D)** MicroRNAs were predicted to target the PIP5K1C 3'UTR via **(C)** TargetScanHuman or **(D)** miRTargetLink 2.0. **(E-F)** Crizotinib-resistant ALK(+) ALCL cells (COSTR200) were transfected miR_25-3p mimic (mimic-miR25-3p), a miR-25-3p inhibitor (inhibit-miR25-3p), or the corresponding negative controls (mimic-CTL and inhibit-CTL)**. (E)** Relative expression of miR_25-3p in COSTR200 cells after transfection measured by RT-qPCR. Snord44 was used as an internal control. Data are shown as 2-ΔΔCt relative values. **(F)** Western blot analysis of PIP5K1C protein levels in COSTR200 cells transfected with a miR_25-3p mimic, miR_25-3p inhibitor (inhib), or their respective negative controls. GAPDH served as a loading control. Experiments were performed in triplicate. Data are presented as mean ± SEM. Statistical significance was determined using an unpaired two-tailed Student's t test with Welch's correction**:** $P < 0.05$ (*), $P < 0.001$ (**), ns = not significant.

Supplementary Figure 8. Tumor growth after PIP5K1C inhibition.

A

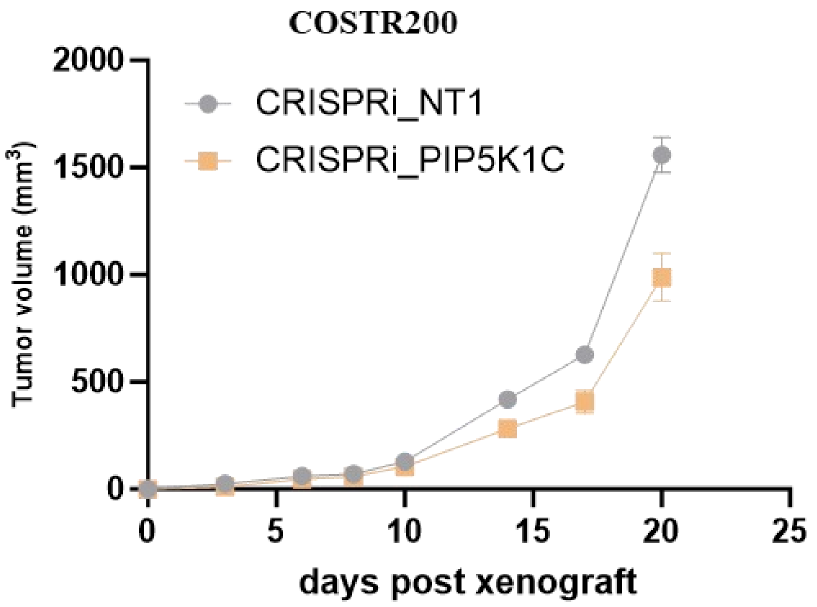

B

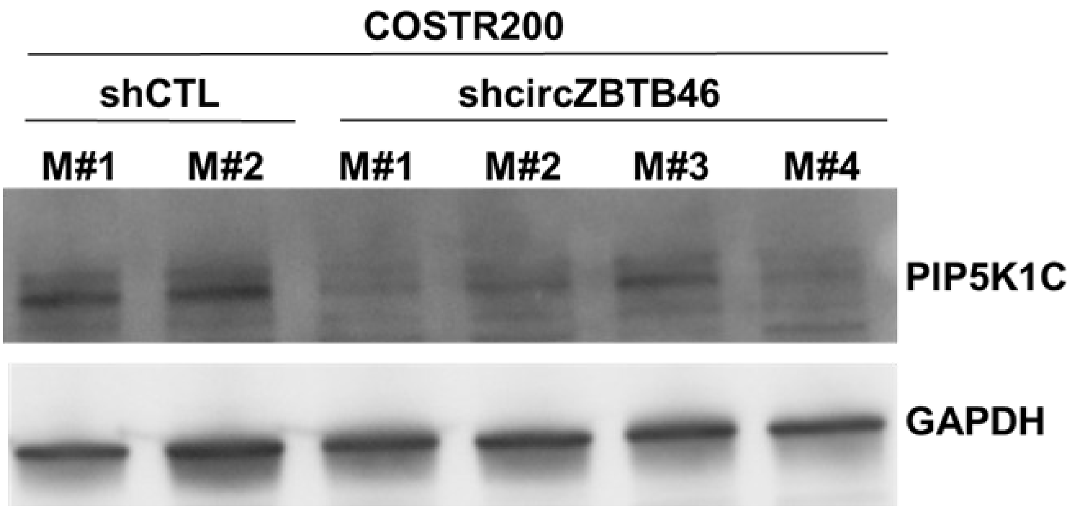

(A) Tumor growth in NSG mice (n=8) injected subcutaneously with COSTR200 cells transduced with PIP5K1C CRISPRi or control CRISPRi and treated with crizotinib (100 mg/kg/day). Tumor volume was monitored over time via calipers and is expressed as the mean ± SEM. **(B)** Western blot analysis of PIP5K1C protein levels in tumors explanted from mice at day 27 initially injected with COSTR200 cells transduced with CTL shRNA or circZBTB46 shRNA. GAPDH served as a loading control.

Supplementary Figure 9. Expression of the *ZBTB46* transcript and circZBTB46 in ALK(+).

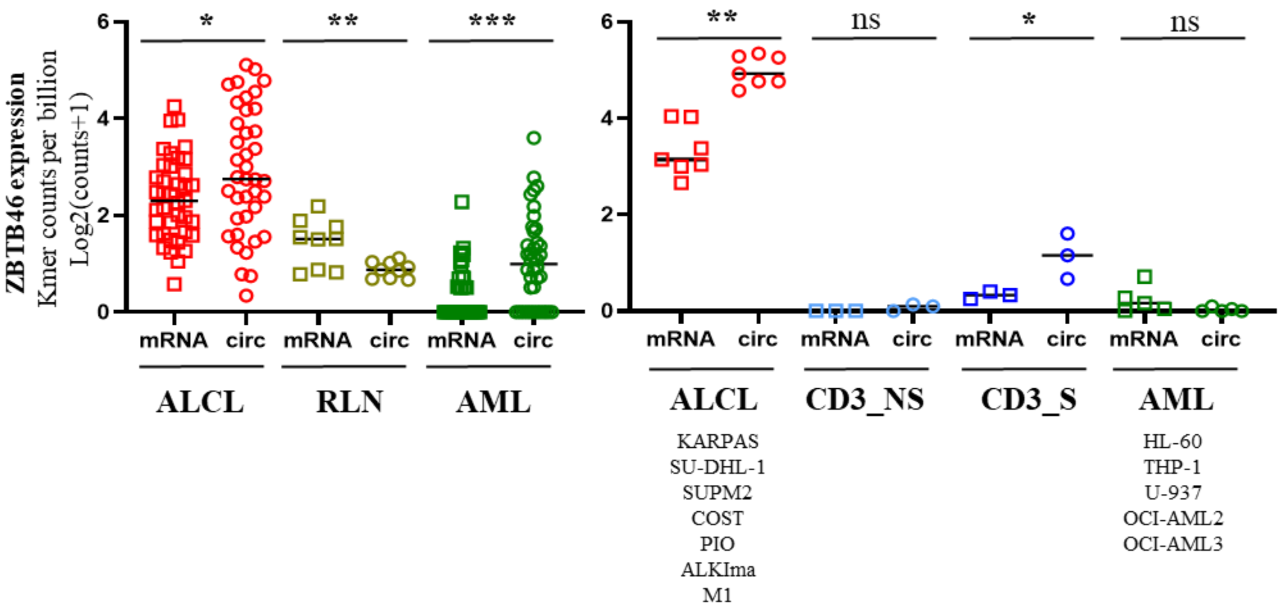

Each dot represents normalized RNA-seq expression (Kmer count per billion [$\log_2$(count+1]) of the indicated RNA in primary biopsy samples, including ALK(+) ALCL (*n* = 39) and AML samples (IUCT-AML cohort (*n* = 40; [11,12], reactive lymph nodes as healthy lymphoid tissue) (RLN, *n* = 9), CD3(+) stimulated (CD3-S, n = 3) or not lymphocytes (CD3-NS, n = 3) and established cell lines (AML, *n* = 5; ALK(+) ALCL, *n* = 7, including in house NPM1::ALK–transformed and immortalized T-cell models (M1: lentiviral NPM1::ALK(+) [13]; 673: CRISPR-Cas9 NPM1::ALK(+)[1]; see Supplementary Figure 2). Statistical significance was assessed via an unpaired two-tailed Student's t test with Welch's correction: $P < 0.05$ (*), $P < 0.01$ (**), $P < 0.001$ (***), ns = not significant. Data are expressed as the means ± SEMs.

Supplementary Table legends:

Supplementary Table S1: Patient sample plan. Samples included in the Ribo-minus RNA sequencing analysis. Reactive lymph nodes (RLN) served as controls (healthy tissue). ALK-positive ALCL cases were classified as relapsed (R) or non-relapsed (NR).

Supplementary Table S2: Circular RNA expression in patient samples. Circular RNAs identified by CIRI2 and analyzed for differential expression using DESeq2 between RLN and ALCL samples. Only significantly differentially expressed circular RNAs are reported. The *Gene_name* column indicates genomic localization (hg38). Normalized read counts are shown for each sample.

Supplementary Table S3: circZBTB46 sequence. Fasta sequence for circular RNA ZBTB46

Supplementary Table S4: Differentially expressed genes after siRNA-mediated knockdown of circZBTB46. Results of analysis following mRNA alignment with HISAT2, read quantification with featureCounts, and differential expression analysis using DESeq2 between siCTL and siCircZBTB46 samples. Only significantly differentially expressed genes are reported. Normalized read counts are provided for each sample.

Supplementary Table S5: miRNA raw count after circZBTB46 pulldown. Raw counts of all microRNAs identified in the circZBTB46 pulldown experiment.

Supplementary Table S6: Enriched miRNA following circZBTB46 pulldown. Differential enrichment analysis was performed using DESeq2 between CTL and circZBTB46 probes. Normalized read counts for each sample are shown.

Supplementary Table S7: nucleotides sequence. Nucleotides sequences for primers, probes, siRNA, shRNA, Kmer used in this study.

References

1. Daugrois C, Bessiere C, Dejean S, Anton-Leberre V, Commes T, Pyronnet S, et al. Gene Expression Signature Associated with Clinical Outcome in ALK-Positive Anaplastic Large Cell Lymphoma. Cancers (Basel). 2021;13(21).

2. Congras A, Hoareau-Aveilla C, Caillet N, Tosolini M, Villarese P, Cieslak A, et al. ALK-transformed mature T lymphocytes restore early thymus progenitor features. J Clin Invest. 2020;130(12):6395-408.

3. Bray NL, Pimentel H, Melsted P, Pachter L. Near-optimal probabilistic RNA-seq quantification. Nat Biotechnol. 2016;34(5):525-7.
4. Soneson C, Love MI, Robinson MD. Differential analyses for RNA-seq: transcript-level estimates improve gene-level inferences. F1000Res. 2015;4:1521.
5. Fuchs S, Babin L, Andraos E, Bessiere C, Willier S, Schulte JH, et al. Generation of full-length circular RNA libraries for Oxford Nanopore long-read sequencing. PLoS One. 2022;17(9):e0273253.
6. Kechin A, Boyarskikh U, Kel A, Filipenko M. cutPrimers: A New Tool for Accurate Cutting of Primers from Reads of Targeted Next Generation Sequencing. J Comput Biol. 2017;24(11):1138-43.
7. Li H, Durbin R. Fast and accurate long-read alignment with Burrows-Wheeler transform. Bioinformatics. 2010;26(5):589-95.
8. Gao Y, Wang J, Zhao F. CIRI: an efficient and unbiased algorithm for de novo circular RNA identification. Genome Biol. 2015;16(1):4.
9. Gao Y, Zhang J, Zhao F. Circular RNA identification based on multiple seed matching. Briefings in Bioinformatics. 2017;19(5):803-10.
10. Love MI, Huber W, Anders S. Moderated estimation of fold change and dispersion for RNA-seq data with DESeq2. Genome Biol. 2014;15(12):550.
11. Zhang Y, Park C, Bennett C, Thornton M, Kim D. Rapid and accurate alignment of nucleotide conversion sequencing reads with HISAT-3N. Genome Res. 2021;31(7):1290-5.
12. Liao Y, Smyth GK, Shi W. featureCounts: an efficient general purpose program for assigning sequence reads to genomic features. Bioinformatics. 2014;30(7):923-30.
13. Potla P, Ali SA, Kapoor M. A bioinformatics approach to microRNA-sequencing analysis. Osteoarthr Cartil Open. 2021;3(1):100131.
14. Menotti M, Ambrogio C, Cheong TC, Pighi C, Mota I, Cassel SH, et al. Wiskott-Aldrich syndrome protein (WASP) is a tumor suppressor in T cell lymphoma. Nat Med. 2019;25(1):130-40.
